# Pathogen diversity drives the evolution of generalist MHC-II alleles in human populations

**DOI:** 10.1371/journal.pbio.3000131

**Published:** 2019-01-31

**Authors:** Máté Manczinger, Gábor Boross, Lajos Kemény, Viktor Müller, Tobias L. Lenz, Balázs Papp, Csaba Pál

**Affiliations:** 1 Synthetic and Systems Biology Unit, Institute of Biochemistry, Biological Research Centre, Hungarian Academy of Sciences, Szeged, Hungary; 2 Department of Dermatology and Allergology, University of Szeged, Szeged, Hungary; 3 MTA-SZTE Dermatological Research Group, University of Szeged, Szeged, Hungary; 4 Institute of Biology, Eötvös Loránd University, Budapest, Hungary; 5 Research Group for Evolutionary Immunogenomics, Max Planck Institute for Evolutionary Biology, Plön, Germany; The Pennsylvania State University, UNITED STATES

## Abstract

Central players of the adaptive immune system are the groups of proteins encoded in the major histocompatibility complex (MHC), which shape the immune response against pathogens and tolerance to self-peptides. The corresponding genomic region is of particular interest, as it harbors more disease associations than any other region in the human genome, including associations with infectious diseases, autoimmune disorders, cancers, and neuropsychiatric diseases. Certain MHC molecules can bind to a much wider range of epitopes than others, but the functional implication of such an elevated epitope-binding repertoire has remained largely unclear. It has been suggested that by recognizing more peptide segments, such promiscuous MHC molecules promote immune response against a broader range of pathogens. If so, the geographical distribution of MHC promiscuity level should be shaped by pathogen diversity. Three lines of evidence support the hypothesis. First, we found that in pathogen-rich geographical regions, humans are more likely to carry highly promiscuous MHC class II DRB1 alleles. Second, the switch between specialist and generalist antigen presentation has occurred repeatedly and in a rapid manner during human evolution. Third, molecular positions that define promiscuity level of MHC class II molecules are especially diverse and are under positive selection in human populations. Taken together, our work indicates that pathogen load maintains generalist adaptive immune recognition, with implications for medical genetics and epidemiology.

## Abstract 2

Whereas specialist major histocompatibility complex (MHC) molecules initiate immune response against only relatively few pathogens, generalists provide protection against a broad range. Accordingly, this study shows that the geographical distribution of generalist MHC alleles in human populations reflects exposure to diverse infectious diseases.

## Author summary

Variation in the human genome influences our susceptibility to infectious diseases, but the causal link between disease and underlying mutation often remains enigmatic. Major histocompatibility complex II (MHC class II) molecules shape both our immune response against pathogens and our tolerance of self-peptides. The genomic region that encodes MHC molecules is of particular interest, as it is home to more genetic disease associations than any other region in the human genome, including associations with infectious diseases, autoimmune disorders, cancers, and neuropsychiatric diseases. Here, we propose that MHC class II molecules can be categorized into two major types; specialists initiate effective immune response against only relatively few pathogens, while generalists provide protection against a broad range of pathogens. As support, we demonstrate that generalist MHC class II variants are more prevalent in human populations residing in pathogen-rich areas.

## Introduction

The major histocompatibility complex (MHC) genes in vertebrates encode cell surface proteins and are essential components of adaptive immune recognition [1]. MHC proteins are endowed with highly variable peptide-binding domains that bind short protein fragments. The MHC region is one of the most polymorphic gene clusters in vertebrate genomes [2]. Co-evolutionary arms race with pathogens is considered largely responsible for the observed exceptionally high levels of genetic diversity [3–6], yet it cannot fully account for the observed geographic differences in human MHC genetic diversity [7, 8]. This indicates that, beyond MHC allelic diversity, other MHC-related factors contribute to the capacity of human populations to withstand pathogens. In this paper, we argue that peptide-binding repertoire size (or, shortly, promiscuity) of MHC alleles is one important factor.

Recent empirical studies demonstrated that there is a substantial variation in the size of the bound and presented antigen repertoire across MHC class I alleles. Certain MHC class I alleles appear to be promiscuous and are capable of binding an exceptionally large set of epitope peptide segments [9, 10]. For example, Paul and colleagues carried out bioinformatics analysis to predict the binding capacity of common HLA-A and HLA-B alleles to a set of 30,000 dengue virus–derived peptides [11]. The analysis revealed over 16-fold variation in the number of peptides bound by the different alleles, indicating significant variation in epitope repertoire size across HLA molecules. The authors selected three alleles for further study in an in vivo transgenic mouse model. Immunization of the corresponding HLA transgenic mouse strains with a set of dengue virus–derived peptides revealed a positive relationship between epitope repertoire size and immunogenicity. Similarly, Kosmrlj and colleagues computed the fraction of self-peptides that bind to various HLA-B molecules, and found that this fraction varies extensively across four HLA-B alleles [12]. The authors then demonstrated that the self-peptide–binding repertoire of HLA-B shapes the native repertoire of T-cell clones developed in the thymus, with implications for recognizing human immunodeficiency virus (HIV) epitopes. Their results could explain why individuals carrying HLA-B*57 alleles can maintain low HIV RNA without therapy. Remarkably, analogous MHC class I alleles with the HLA-B*27 superfamily is widespread in Chinese rhesus macaques, animals which show especially slow progression of simian immunodeficiency virus (SIV)/HIV [13]. Finally, by focusing on seven chicken MHC class I haplotypes and four human HLA-B alleles, Chappel and colleagues demonstrated that MHC class I molecules that can bind a wide range of viral epitopes show lowered expression on the cellular surfaces of immune cells, such as monocytes and lymphocytes [9]. The authors suggested that the breadth of epitope-binding repertoire shapes genetic susceptibility to Marek’s disease virus in chickens and HIV disease progression in humans.

More generally, by recognizing more peptide segments, promiscuous MHC molecules may promote immune response against a broader range of pathogens and are hence generalists [9, 10]. Prior case studies in chicken indicate that this may be so [14–17]. However, it remains to be established whether this relationship generally holds across MHC class I and II alleles and a wide range of infectious diseases. Specifically, we propose that in regions of high pathogen diversity, human populations should carry promiscuous MHC alleles. Moreover, as migrating human populations have been exposed to changing sets of pathogens [18], shifts in MHC promiscuity level should have occurred repeatedly and in a rapid manner during the course of human evolution.

To test these predictions, we first focused on the human HLA class II DRB1 gene, for several reasons. First, DRB1 is the most variable HLA class II locus, with over 2,000 registered alleles [19]. Together with HLA-DRA, HLA-DRB1 encodes the heterodimeric HLA-DR protein complex, but HLA-DRA is basically invariant. Second, DRB1 shows the strongest general signature of selection among HLA class II loci [20], while at the same time showing the weakest evidence for divergent allele advantage, an alternative mechanism at the genotype level for presenting a broader set epitopes [21]. Third, DRB1 has diversified very rapidly in the human lineage [22]. Many of the DRB1 alleles appear to be human specific and most likely evolved after the migration of ancestral human populations out of Africa [22]. These periods have been associated with human populations encountering numerous new pathogens [18, 23]. For other HLA class II loci, the level of genetic diversity is lower [19, 24], probably driven by selection for functions partly unrelated to pathogens. Notably, HLA-DQ has a fundamental role in the development of immune tolerance [25, 26], while HLA-DP contributes to the presentation of epitopes of intracellular origins [27–29]. Fourth, epitope-binding prediction algorithms show higher accuracy for DRB1 than for other HLA class II loci [30, 31]. Finally, the abundance of DRB1 on the cell surface is especially high compared with other HLA class II molecules [32–34]. Subsequently, we also evaluated promiscuity patterns of HLA class I molecules.

Estimates on epitope-binding promiscuity were derived from two sources: experimental assays that measured individual peptide–MHC interactions in vitro and systematic computational predictions. In a series of analyses, we show that predictions of our hypothesis are upheld, regardless of how HLA-DRB1 promiscuity level is estimated.

## Results

### Estimating HLA-DRB1 promiscuity level

Given that large-scale experimental assays to measure individual peptide–MHC interactions are extremely tedious, we first employed established bioinformatics tools to predict the binding affinities of experimentally verified epitope peptides for a panel of 162 nonsynonymous HLA-DRB1 alleles, all of which are present at detectable frequencies in at least one human population [35–37]. The set of investigated epitopes was derived from the Immune Epitope Database (IEDB) and contains 2,691 peptide epitopes of 71 pathogens known to be bound by certain HLA class II alleles [38] (S1 Data). Epitopes showing high levels of amino acid similarity to each other were excluded from the analysis (See Methods). Most included epitopes are 15 to 20 amino acids long and are found in only one of the 71 pathogens (S1 Fig, S1 Data). The NetMHCIIpan algorithm was used to predict individual epitope–MHC interactions [30], not least because it outperforms other prediction algorithms [31]. The breadth of epitope-binding repertoire or, shortly, the level of promiscuity of individual HLA-DRB1 alleles was estimated as the fraction of epitopes with a binding affinity stronger than 50 nM to the given MHC molecule. This threshold corresponds to high-affinity binding, which is frequently observed in MHC molecules displaying immunodominance [39]. We found large variation in promiscuity levels across HLA-DRB1 alleles (S2 Fig, S2 Data). Using a smaller dataset with information from both approaches, we show that the computationally predicted and the empirically estimated promiscuity values are strongly correlated with each other (Spearman’s rho: 0.78, *P* = 0.004, S3 Fig). Moreover, our results are robust to changes in the affinity threshold (S4A to S4C Fig), usage of other prediction algorithms (S4D Fig), and variation in the epitope data employed (S4E Fig). As expected, promiscuous HLA-DRB1 alleles can present epitopes from a broader range of pathogen species (S5 Fig). Reassuringly, there was no correlation between allele promiscuity values and the amount of data per allele used for the training of the algorithm (Spearman’s rho: −0.38, *P* = 0.21).

### Global distribution of promiscuous HLA-DRB1 alleles

Taking advantage of the confirmed reliability of computational predictions, we next investigated the geographic distribution of HLA-DRB1 alleles. We first collected high-quality HLA-DRB1 allele prevalence data of 96 human populations residing in 43 countries from two databases and an article [35–37]. The weighted average of promiscuity level in each population was calculated based on the promiscuity values and allele frequencies of individual alleles in the population (See Methods). The analysis revealed a large variation in mean promiscuity across geographical regions and the corresponding human populations (S1 Table). Importantly, several distantly related but highly promiscuous alleles contribute to this pattern (S1 Table). Notably, an especially high allelic promiscuity level was found in Southeast Asia, an important hot spot of emerging infectious diseases [40]. To minimize any potential confounding effect of high genetic relatedness between neighboring populations, we merged populations with similar HLA allele compositions for all further analyses (See Methods).

### Link between pathogen diversity and HLA-DRB1 promiscuity level

Using the Global Infectious Diseases and Epidemiology Network (GIDEON), we compiled a dataset on pathogen richness in the corresponding 43 geographic regions [41]. It consists of 95 diseases caused by 168 extracellular pathogens, including diverse bacterial species, fungi, protozoa, and helminthes. Using the same protocol, we additionally compiled a dataset on the prevalence of 149 diseases in the same regions caused by 214 viral and other obligate intracellular pathogens. The dataset and methodology employed for the analysis are standardized and have been used previously in similar contexts [7, 8, 42].

**Fig 1:**
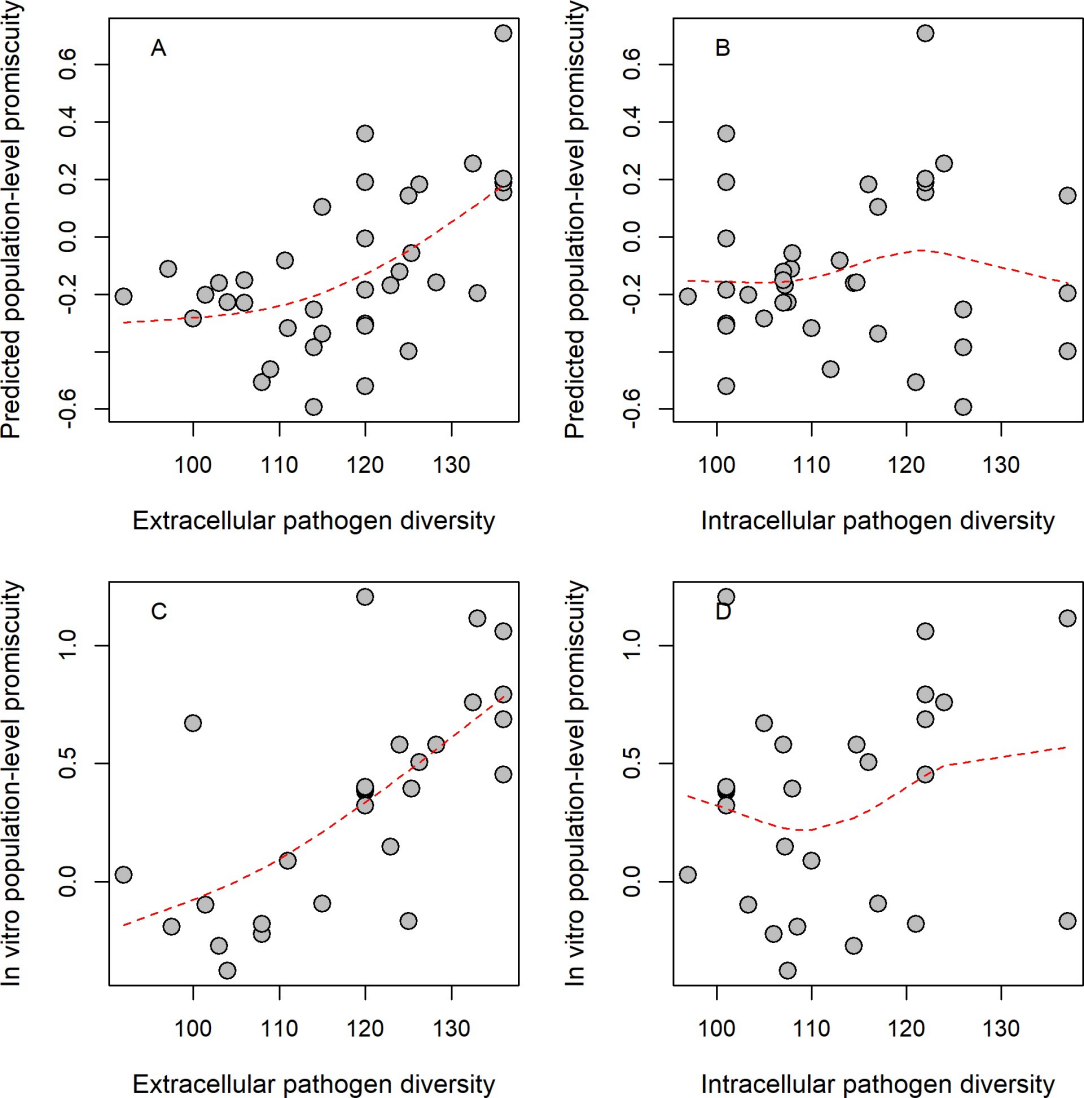
Relationship between epitope-binding promiscuity and pathogen diversity. Normalized population-level promiscuity of HLA-DRB1 alleles is shown as the function of extracellular pathogen diversity, as approximated by species count. Promiscuity scores were calculated based on standardized (i.e., z-score) allele promiscuity values and were averaged in each population group (see Methods). Significant correlations were found between extracellular pathogen species count and **(A)** predicted allele promiscuity level in 37 groups (Spearman’s rho: 0.5, *P* = 0.002) and **(C)** in vitro promiscuity level in 28 groups (Spearman’s rho: 0.7, *P* = 3*10^−5^). No significant correlation was found between intracellular pathogen diversity and **(B)** predicted and **(D)** in vitro promiscuity at the HLA-DRB1 locus (Spearman’s rho: 0.04 and 0.21, *P* = 0.81 and 0.29, respectively). Dashed lines indicate smooth curve fitted using cubic smoothing spline method in R (see Methods). Population groups were created using the 15th percentile genetic distance cutoff (see Methods). For results obtained upon using alternative distance cutoff values, see S3 Data. The underlying data for this figure can be found in S4 Data.

We report a strong positive correlation between extracellular pathogen diversity and mean promiscuity: HLA-DRB1 alleles that can bind epitopes from a broader range of pathogens are more likely to be found in regions of elevated pathogen diversity (Fig 1A). This pattern is unlikely to be explained by confounding factors, such as country size or HLA-DRB1 genetic diversity across countries (S2 Table). By contrast, we found no significant association between HLA-DRB1 promiscuity level and diversity of intracellular pathogens (Fig 1B). We conclude that the geographical distribution of promiscuous HLA-DRB1 alleles has been mainly shaped by the diversity of extracellular pathogens.

**Fig 2:**
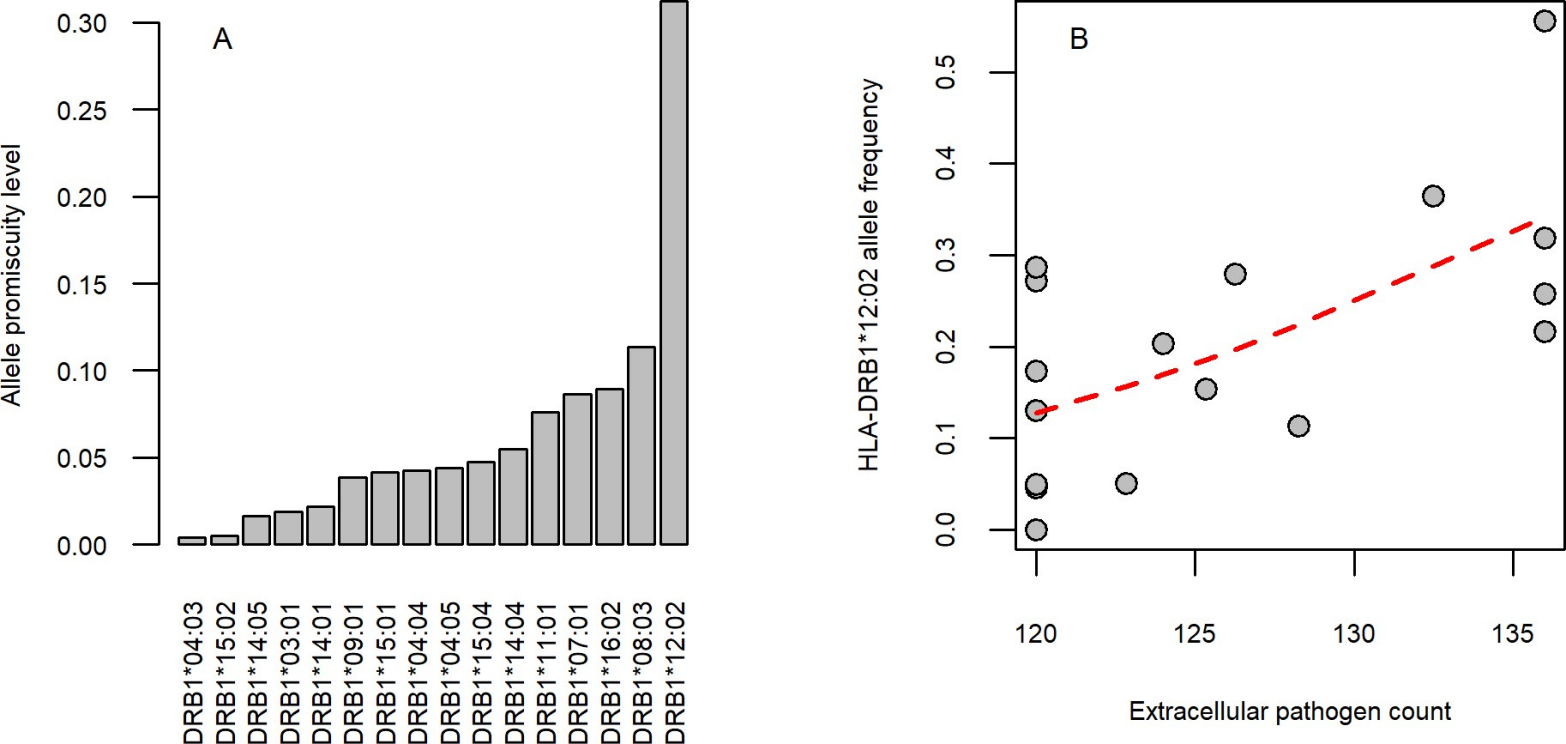
HLA-DRB1*12:02 allele promiscuity level and extracellular pathogen diversity in Southeast Asia. **(A)** HLA-DRB1*12:02 has an exceptionally high promiscuity level compared with other alleles. The figure shows alleles with at least 10% frequency in at least one population in Southeast Asia. Predicted allele promiscuity values are shown. **(B)** The mean frequency of DRB1*12:02 increases with extracellular pathogen diversity across population groups (Spearman’s rho: 0.57, *P* = 0.017). Populations that resided in China, Japan, South Korea, Indonesia, Malaysia, and the Philippines were included in the analysis. Red curve indicates smooth curve fitted using cubic smoothing spline method in R (see Methods). The underlying data for this figure can be found in S4 Data.

The above results hold—and are even stronger—when estimates on promiscuity were derived from empirical in vitro MHC binding data (shortly, in vitro promiscuity), downloaded from the IEDB database [38] (Fig 1C and 1D, S2 Table and S3 Data). However, these results do not exclude the possibility that the geographical link between pathogen diversity and promiscuity is indirect. More direct support on the causal relationship between the two variables comes from analysis of prior human genetic studies. To investigate this issue, we focused on two geographically widespread allelic groups with exceptionally high (HLA-DRB1*12) and exceptionally low (HLA-DRB1*03) promiscuity values, respectively, and conducted literature mining on their reported associations with infectious diseases (S3 Table). As expected, HLA-DRB1*12 was associated with protection against at least five infectious diseases, while HLA-DRB1*03 was associated with susceptibilities to eight infectious diseases, which is highly unlikely by chance (Fisher test, *P* = 0.003) (S3 Table, S5 Data). The data also indicate local adaptation towards elevated promiscuity under diverse pathogen pressure. The HLA-DRB1*12:02 allele is prevalent in specific regions of Southeast Asia. Compared with other alleles detected in this region, HLA-DRB1*12:02 has a relatively high promiscuity value (top 20%, Fig 2A, S2 Data). The high frequency of HLA-DRB1*12:02 has been previously suggested to reflect pathogen-driven selection during the migration of a Mongolian population to South China [43]. Indeed, this allele is associated with protection from recurrent pulmonary tuberculosis, recurrent typhoid fever, and hepatosplenic schistomiasis (S5 Data), all of which are endemic diseases in Southeast Asia [44–46]. Remarkably, the frequency of this allele increases with extracellular pathogen diversity in this region (Fig 2B). Together, these observations support the hypothesis that promiscuous epitope binding of HLA-DRB1 alleles is favored by selection when extracellular pathogen diversity is high.

### Evolution of promiscuous HLA alleles

**Fig 3:**
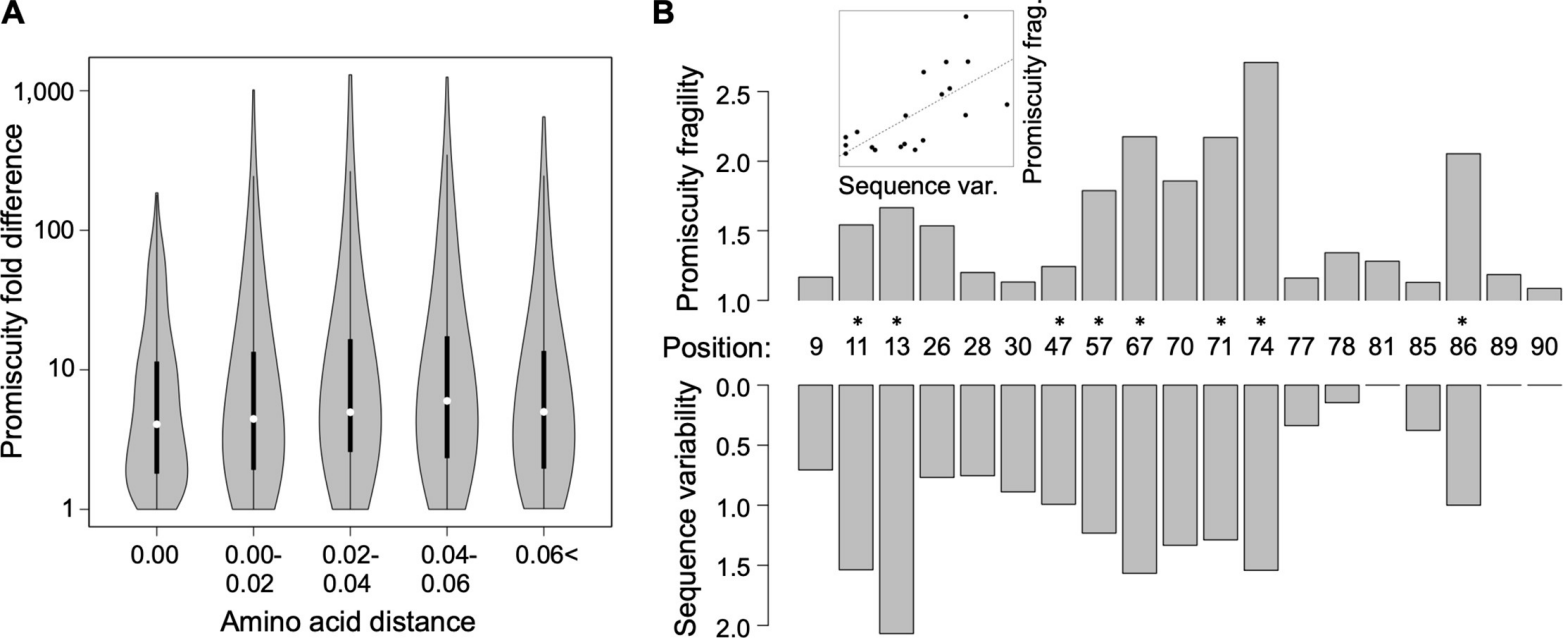
Promiscuity changes rapidly during evolution and might be a selectable trait. **(A)** For all pairs of selected alleles, the predicted promiscuity difference between two HLA-DRB1 alleles is shown as a function of amino acid distance measured after excluding the epitope-binding region. Large differences in promiscuity can be observed even between closely related pairs of alleles (e.g., at zero amino acid distance). As a result, there is no correlation between amino acid distance and promiscuity fold difference (Spearman’s rho = 0.02, *P* = 0.19). Amino acid distances were binned as shown on the figure (*n* = 308, 1,168, 564, 654, 1,492). Violin plots show the density function of promiscuity fold difference values for allele pairs in the given bin. White circles show median values; bold black lines show the interquartile range. **(B)** Sequence variability of an amino acid site in the epitope-binding region of HLA-DRB1 (measured as Shannon entropy) correlates positively with the site’s promiscuity fragility, measured as the median predicted promiscuity fold difference caused by a random amino acid change at the given site (see inset, Spearman’s rho: 0.76, *P* = 0.0001). Sites that have a larger impact on promiscuity are more diverse in human populations. Line in inset represents linear regression between the two variables. The same result was obtained when promiscuity fragility was calculated based on nucleotide substitutions instead of amino acid substitutions (Spearman’s rho: 0.73, *P* = 0.0004, see Methods) or when sequence variability was measured as nonsynonymous nucleotide diversity (π_A_) instead of sequence entropy (S7 Fig). Sites under positive selection as identified by Furlong and colleagues [48] show significantly higher promiscuity fragility (Wilcoxon rank sum test, *P* = 0.0012) and are marked with asterisks (see also S8 Fig). The underlying data for this figure can be found in S4 Data.

An important unresolved issue is how promiscuity has changed during the course of human evolution. Under the assumption that local pathogen diversity drives the evolution of epitope recognition of HLA class II alleles, promiscuity as a molecular trait should have evolved rapidly as human populations expanded into new territories. To investigate this issue, we combined an established phylogeny of HLA-DRB1 alleles [47] with predicted epitope-binding promiscuity values. We found that alleles with a high promiscuity level have a patchy distribution across the tree (S6 Fig), indicating that high promiscuity has multiple independent origins. To investigate this observation further, we selected a set of 96 HLA-DRB1 alleles with a detectable frequency in at least one human population and appropriate sequence data (see Methods). A comparison of all pairs of these alleles revealed that even very closely related alleles show major differences in promiscuity levels (Fig 3A). For example, alleles belonging to the HLA-DRB1*13 group show over 98% amino acid sequence identity to each other, but display as much as 57-fold variation in the predicted promiscuity levels. We conclude that the switch between high and low promiscuity levels has occurred repeatedly and in a rapid manner during the allelic diversification of the HLA-DRB1 locus.

We next asked how selection on promiscuity has shaped the genetic diversity along the epitope-binding region of HLA molecules. To quantify protein sequence variability at each amino acid position, we calculated the Shannon entropy index based on the alignment of the 96 selected HLA-DRB1 alleles from above. For each position, we also calculated promiscuity fragility, that is, the median impact of single amino acid substitutions on promiscuity (see Methods). A strong positive correlation was found between Shannon entropy and promiscuity fragility (Fig 3B, Spearman’s rho = 0.76, *P* = 0.0001). Importantly, this conclusion does not depend on how sequence polymorphism was estimated (S7 Fig). Accordingly, amino acid positions with a large impact on epitope-binding promiscuity are highly variable in human populations. Furthermore, those sites in the epitope-binding region that are under positive selection [48] tend to have high promiscuity fragility values (Wilcoxon rank sum test, *P* = 0.0012, Fig 3B; see also S8 Fig). The above data suggest a link between allele promiscuity and HLA-DRB1 diversification, probably as an outcome of selection for locally optimized promiscuity levels.

Finally, we note that several variable molecular sites in the binding region of HLA-DRB1 affect epitope-binding characteristics without any major impact on promiscuity per se. For example, our computational analysis indicates that mutations at amino acid site numbers 9 and 47 do not seriously affect promiscuity level (Fig 3B). However, several mutations at these sites are associated with binding self-peptides and thereby shape vulnerability to specific autoimmune diseases [49, 50].

### Pathogen diversity and HLA class I promiscuity

The relationship between pathogen diversity and epitope-binding promiscuity may be more general, as similar results hold for the HLA-A locus. HLA-A is one of the three types of classical human MHC class I molecules and is mainly involved in the presentation of epitopes from intracellular pathogens [51]. In agreement with expectation, we report a positive correlation between local intracellular pathogen diversity and the HLA-A promiscuity level of the corresponding human populations (S9A and S9B Fig, S3 Data). No marked positive correlation was found for two other MHC class I genes (HLA-B and HLA-C, see S9C to S9F Fig, and S3 Data). Therefore, other unrelated evolutionary forces may shape the geographical distribution of promiscuous HLA-B and HLA-C alleles (S1 Text).

## Discussion

Central players of the adaptive immune system are the groups of proteins encoded in the MHC. By binding short peptide segments (epitopes), MHC molecules guide both immune response against pathogens and tolerance to self-peptides. The genomic region encoding these MHC molecules is of special interest, for two reasons. It harbors more disease associations than any other regions in the human genome, including associations with infectious diseases, autoimmune disorders, tumors, and neuropsychiatric diseases [52, 53]. A growing body of literature is now revealing that certain MHC class I alleles can bind a wider range of epitopes than others, but the functional implications of this variation remain largely unknown [10]. By recognizing a larger variety of epitopes, such promiscuous MHC alleles promote immune response against a broader range of pathogens at the individual level. Therefore, promiscuous epitope binding of MHC molecules should be favored by selection in geographic regions where extracellular pathogen diversity is high. Importantly, this mechanism is conceptually distinct from the well-established concept of heterozygote advantage at the MHC [54], as it concerns individual alleles and not allele combinations or genotypes.

To test this hypothesis, we combined data on the geographic distribution of human MHC class II alleles and prevalence of extracellular pathogens, empirical/computational estimates of epitope-binding promiscuity, and phylogenetic analyses. Our main findings, strongly supporting our hypothesis, are as follows.

First, in geographical regions of high extracellular pathogen diversity, human HLA-DRB1 alleles have exceptionally high epitope-binding repertoires. This suggests that the geographical distribution of promiscuous HLA-DRB1 alleles has been shaped by the diversity of extracellular pathogens. The HLA-DRB1*12:02 allele highlights this point. HLA-DRB1*12:02 is a promiscuous allele that has been associated with protection from certain infectious diseases (S5 Data). As expected, this allele is especially prevalent in regions of Southeast Asia with elevated pathogen load (Fig 2B).

It is well established that antigens presented by HLA class II molecules derive mainly from extracellular proteins [1]. However, HLA class II molecules have well-established roles in controlling immune response against viruses [55, 56]. Additionally, viral peptides are reported to be processed and presented also by the HLA class II pathway [57]. Therefore, it remains to be established why intracellular pathogen diversity has no major impact on the global distribution of HLA-DRB1 alleles.

Notably, the relationship between pathogen load and epitope-binding promiscuity may be more general, as similar results hold for the HLA-A locus: we found a positive correlation between local intracellular pathogen diversity and the HLA-A promiscuity level of the corresponding human populations (S9A and S9B Fig, S3 Data).

Second, a phylogenetic analysis revealed major differences in promiscuity levels of very closely related HLA-DRB1 alleles. This suggests that high promiscuity level in HLA-DRB1 has evolved rapidly and repeatedly during human evolution. Finally, amino acid positions with a prominent role in shaping HLA-DRB1 promiscuity level are especially variable in human populations and tend to be under positive selection. In sum, we conclude that HLA promiscuity level is a human trait with paramount importance during adaptation to local pathogens.

Our work has important ramifications for future studies. MHC is the most variable region of the human genome, and the variation is associated with numerous infectious and immune-mediated diseases [52, 53, 58–62]. The impact of MHC promiscuity level on population allelic diversity is an interesting area for future research. In a similar vein, MHC allelic diversity is associated with olfaction-based mating preferences in human and other animals [63]. The roles of MHC promiscuity in mating success and mating preferences are a terra incognita.

We note that the most promiscuous HLA-DRB1 alleles are rare in certain human populations (S1 Table; S2 Data). This suggest that these alleles are not particularly favored by natural selection in these areas. Why should it be so? First, high promiscuity may not be able to cope with the rise of novel and highly virulent pathogens. In such cases, displaying a particular epitope might be the most efficient way to achieve resistance, and high promiscuity might be suboptimal due to a reduced specificity [9, 10]. Second, high promiscuity level may elevate the risk of immune reactions against host tissues and non-harmful proteins [9, 64]. Clearly, future work should elucidate the evolutionary trade-offs between protection from pathogens and genetic susceptibility to autoimmune diseases. This will require high-throughput experimental methods to determine epitope-binding repertoire [65], and HLA transgenic mice studies on the role of promiscuity in immune response [66].

Finally, genetic variation within particular MHC genes influences vaccine efficacy [67], rejection rates of transplanted organs [68], susceptibility to autoimmune diseases [49], and antitumor immunity [28, 69, 70]. Our work raises the possibility that, by altering the maturation and functionality of the immune system, the size of the epitope-binding repertoire of MHC alleles itself could have an impact on these processes. The exact role of MHC promiscuity in these crucial public health issues is an exciting future research area.

## Methods

### Computational prediction of epitope-binding promiscuity

The IEDB has collected the results of individual and systematic studies on epitope binding by MHC alleles [38]. The experimental studies include HLA-binding assays, T-cell activation assays, and immunopeptidomic studies as well. Epitopes of all available viral, bacterial, and eukaryotic pathogens known to be bound by at least one HLA-I or HLA-II allele were extracted from IEDB. Reference proteomes of pathogenic species that carry at least one of the collected epitope sequences were retrieved from the Uniprot database (102 for HLA-I and 71 for HLA-II epitopes) [71]. Only epitopes of these species were analyzed further. All proteomes were scanned for each epitope sequence, and epitope sequences found in only one proteome (i.e., species-specific epitopes) were kept for further analysis. Highly similar epitope sequences were identified using Clustal Omega [72] and excluded as follows. A protein distance matrix was created and epitopes were discarded iteratively. In each iteration, the epitope pairs with the lowest k-tuple distance were identified. Then, the epitope with the highest average similarity to all other sequences was excluded. Iterations were repeated until distance values less than 0.5 (corresponding to greater than approximately 50% sequence identity) were eliminated from the matrix [73]. Note that this filtering procedure was carried out separately for epitope sequences bound by HLA-I and HLA-II.

Binding affinities of the remaining 3,265 HLA-I epitope sequences to 346 HLA-A, 532 HLA-B and 225 HLA-C alleles were predicted with the NetMHCpan-4.0 algorithm [74]. The binding of 2,691 HLA-II epitope sequences to 162 HLA-DRB1 alleles was predicted using the NetMHCIIpan-3.1 algorithm [30]. All 162 alleles are present in at least one of the human populations studied here (see below). The “pep” sequence input format was used for both HLA-I and HLA-II epitope-binding prediction. A binding affinity threshold of 50 nM was applied, yielding peptides that are likely to be immunodominant [39]. For alternative binding threshold definitions, see S4 Fig. For each binding threshold, epitope-binding promiscuity was defined as the fraction of the epitope set bound by a given allele.

### Calculating epitope-binding promiscuity using in vitro data

To determine the epitope-binding promiscuity of HLA-DRB1 alleles based on previously published experimental data, we used the IEDB database [38]. Specifically, we downloaded all MHC ligand and T-cell assay data, which was available for 48 HLA-DRB1 alleles. Binding data of 20 alleles screened for at least 100 ligands each were further analyzed. The epitope set of each allele was filtered for highly similar sequences, as described above. As the majority of in vitro assay data were available in a binary format (i.e., presence or absence of binding), promiscuity was calculated as the fraction of positive binding assays for a given allele.

### Calculating promiscuity levels of human populations

To calculate population-level promiscuity values, we obtained HLA allele frequency data from the Allele Frequency Net Database (AFND) and the International Histocompatibility Working Group (IHWG) populations [35, 36]. Haplotype-level data of the 13th International HLA and Immunogenetics Workshop (IHIW) populations were downloaded from dbMHC (National Center for Biotechnology Information [NCBI]; ftp://ftp.ncbi.nlm.nih.gov/pub/mhc/mhc/Final%20Archive). Additionally, allele frequency data of the 14th and 16th IHIW populations, as published by Riccio and colleagues [37], and populations in the AFND were used in the analyses. To avoid potential confounding effects of recent genetic admixture and migration, we focused on native populations, similarly to previous studies (S1 Table) [7, 8]. We excluded IHWG populations reported to deviate from Hardy-Weinberg equilibrium [37]. Among the AFND populations and IHWG populations without haplotype-resolution data (14th and 16th IHIW), those comprising less than 100 genotyped individuals or those in which the sum of allele frequencies deviated from 1 by more than 1% were excluded. Populations reported in multiple databases were included only once in the analysis.

For each HLA loci, we calculated mean population promiscuity by averaging promiscuity values of alleles weighted by their relative frequencies in the populations. In all of these calculations, we used standardized (i.e., z-score) promiscuity values to make the in silico and in vitro values more easily comparable. Finally, when calculating population-level promiscuity based on in vitro promiscuity data, we excluded populations for which in vitro promiscuity values could be assigned to less than 50% cumulative allele frequency.

To tackle the issue of nonindependence of data points, we focused on populations instead of countries and grouped those populations that have highly similar HLA allele compositions, based on standard measures of genetic distance (see below). We merged populations with highly similar HLA allele compositions, allowing us to avoid pseudoreplication of data points while retaining informative allele frequency differences between populations residing in the same broad geographical areas.

To this end, we first generated a genetic distance matrix between populations with the adegenet R library using allele frequency data of the examined locus. We used the Rogers’ genetic distance measure [75] because it does not assume that allele frequency changes are driven by genetic drift only, an unlikely scenario for HLA genes. Next, populations were merged using a network-based approach. Populations were treated as nodes and two nodes were connected if their genetic distance was under a cutoff value. Populations were grouped in an iterative manner. In each iteration, all maximal cliques (i.e., subsets of nodes that are fully connected to each other) in the network were identified. Maximal cliques represent groups of populations in which all populations have similar allele compositions to each other. Then, mean genetic distance within each clique was calculated. The clique with the lowest average distance was selected and its populations were grouped together. Then, this clique was deleted from the network. Iterations were repeated until no maximal cliques remained in the network. Grouping of populations was carried out using different distance value cutoffs (1st, 5th, 10th, and 15th rank percentile of all distance values). The resulting population groups and the individual populations that remained in the network were treated as independent data points in subsequent statistical analyses. Mean promiscuity level in population groups was calculated by averaging population promiscuity values.

Unless otherwise indicated, all figures are based on population groups using the 15th percentile genetic distance cutoff value. Importantly, using different cutoffs has no impact on our results (S3 Data). Finally, we note that genetic differences among human populations mostly come from gradations in allele frequencies rather than from the presence of distinctive alleles [76]. Therefore, traditional clustering of populations based on HLA composition would have been ill-suited for our purposes.

### Pathogen diversity

Data on 309 infectious diseases were collected from GIDEON [41]. For each disease, the number of causative species or genera (when species were not listed for the genus) was determined using disease information in the GIDEON database, as described previously [42]. Causative agents were classified into obligate intracellular and extracellular pathogen groups based on a previous study [7] and literature information. Putative facultative intracellular pathogens were excluded from the analysis. Diseases caused by agents that could not be clearly classified were also excluded from the analysis. Extracellular and intracellular pathogen diversity (richness) of each country was approximated by the number of identified endemic extracellular and intracellular species, respectively.

Finally, we assigned country-level measures of pathogen and HLA diversity to population groups as follows. For each population group, extracellular and intracellular pathogen counts were calculated by averaging the corresponding country-level values across the populations within the group. For example, if a population group contained two populations residing in neighboring countries, then we assigned the average pathogen diversity of the two countries to it.

### Literature mining of associations between HLA alleles and infectious diseases

To examine associations between selected HLA allele groups and infectious diseases, we carried out a systematic literature search on PubMed database using the following terms:

“assoc* drb1 12 02”, “assoc* drb1 1202”, “assoc* drb1 12 01”, “assoc* drb1 1201”, “assoc* dr12”, “assoc* drb1*12”, “assoc* drb1 03 01”, “assoc* drb1 0301”, “assoc* dr3”, “assoc* drb1*03”, “assoc* dr17”, “infect* drb1 12 02”, “infect* drb1 1202”, “infect* drb1 12 01”, “infect* drb1 1201”, “infect* dr12”, “infect* drb1*12”, “infect* drb1 03 01”, “infect* drb1 0301”, “infect* dr3”, “infect* drb1*03”, and “infect* dr17”.

“assoc*” and “infect*” refer to any word beginning with these letters.

Each resulting paper containing HLA association data was examined, and statistically significant associations between allele groups (DRB1*03 or DRB1*12) or common alleles in allele groups (DRB1*12:01, DRB1*12:02, DRB1*03:01) and infectious diseases were collected. Associations with diseases caused by intracellular pathogens were excluded from the analysis. HLA-disease associations were classified as beneficial or detrimental, if all related studies supported the beneficial or detrimental role of HLA allele/allele group in the development or course of the given disease. Otherwise, the association was classified as controversial. The results were summarized (S3 Table), and statistical association between beneficial/detrimental effects and high/low promiscuity across allele groups was determined by a Fisher’s exact test.

### Amino acid distance between DRB1 alleles

We used amino acid distance as a proxy for phylogenetic distance between pairs of DRB1 alleles. To this end, nucleotide sequences of DRB1 alleles that contained full exon 2 and 3 regions were downloaded from the IPD-IMGT/HLA database [19]. To limit our analyses to alleles that have an impact on the inferred promiscuity level of a population, we considered only those sequences that had a nonzero frequency in at least one human population (see above). From allele groups that code for the same protein sequence (synonymous differences, differentiated by the third set of digits in the HLA nomenclature), one of the alleles was randomly chosen. This selection procedure resulted in 96 alleles. Multiple alignment of nucleotide sequences was performed using the MUSCLE algorithm as implemented in the MEGA software [77] and converted to protein sequence alignments. Amino acid distance was calculated using the Jones-Taylor-Thornton substitution model in MEGA [77] (Fig 3A). Epitope-binding region sites—as defined previously [30]—were excluded when calculating amino acid distance. The rationale behind this exclusion is that these sites are known to be under positive selection [78, 79] and are therefore less informative on evolutionary distance. Additionally, by removing these sites, the amino acid distance remains independent of promiscuity predictions. Finally, as intragenic recombination may distort the inference of evolutionary distance, we identified such events across all alleles following the protocol of Satta and colleagues [80] using GENECONV [81] and RDP algorithms [82] as implemented in the RDP software [83]. Recombinant alleles were removed when calculating amino acid distance.

### Sequence diversity and promiscuity fragility

We first defined the epitope-binding region of HLA-DRB1 alleles, as previously [30]. To estimate sequence diversity along the epitope-binding region, we employed two measures: standard Shannon entropy [84] and nucleotide diversity (π), a widely employed measure of genetic variation [85].

Using the protein sequence alignment of the 96 alleles defined above, we calculated amino acid sequence variability as the Shannon entropy of the given amino acid site as follows:

$$\sum_{i=1}^{M}P_{i}{log}_{2}P_{i}$$

where P_i_ is the fraction of residues of amino acid type i at a given site, and M is the number of amino acid types observed at that site.

Nonsynonymous nucleotide diversity (*π*_*A*_) measures the average number of nonsynonymous nucleotide differences per nonsynonymous site between two randomly chosen protein coding DNA sequences from the same population [85, 86]. *π*_*A*_ was calculated for each amino acid site in the epitope-binding region for each population using DnaSP software [87] and custom-written R scripts. Nucleotide sequences of DRB1 alleles were downloaded from the IPD-IMGT/HLA database [19].

The calculation is based on the work of Nei and colleagues [85] using the equation

$$\pi_{A}=\sum_{i,j}x_{i}*x_{j}*\pi_{A_{ij}}$$

where x_i_ and x_j_ are the frequencies of the ith and jth alleles in the population, respectively, and $\pi_{A_{ij}}$ is the number of nonsynonymous nucleotide differences per nonsynonymous nucleotide site between the two codon sequences of the given amino acid site in the ith and jth alleles. To calculate *π*_*A*_ for each population, allele frequency data of human populations were obtained, as described earlier (see above). An overall nucleotide diversity index was calculated by averaging *π*_*A*_ across populations.

To calculate each amino acid site’s impact on epitope-binding promiscuity (promiscuity fragility), promiscuity was predicted for each 19 possible amino acid change along the epitope-binding region of each of 96 alleles. The fold difference in promiscuity resulting from each amino acid substitution was calculated. The median promiscuity fold difference of each possible allele and amino acid change combination (96 × 19) was used to estimate promiscuity fragility at each amino acid position. As some of the 19 possible amino acid changes are not accessible via a single nucleotide mutation, and the accessible amino acid changes can have different likelihoods based on the codon sequence of the site and the genetic code, we also calculated promiscuity fragility based on each nonsynonymous nucleotide substitution of the codon instead of each amino acid substitution of the site.

### Statistical analysis and graphical representation

All statistical analyses were carried out in R version 3.2.0 [88]. Smooth curves were fitted using the cubic smoothing spline method [89].

## Supporting information

S1 Fig Length distribution of 2,691 epitopes used for the prediction of HLA-DRB1 allele promiscuity. The underlying data for this figure can be found in S4 Data.
(TIF)

S2 Fig Epitope-binding promiscuity values show large variation across alleles. The plot shows the fraction of epitopes bound by HLA-DRB1 alleles, as predicted computationally. Each allele is present in at least 1% frequency in at least one examined human population. The underlying data for this figure can be found in S4 Data.
(TIF)

S3 Fig Correlation between computationally predicted and in vitro–measured allele promiscuity levels. To verify the accuracy of computational promiscuity predictions, a quantitative in vitro dataset containing 44,541 experimentally measured binding affinity values for 11 HLA-DRB1 alleles was downloaded from the IEDB database [38]. We selected (i) 216 epitope sequences from this dataset, for which binding affinity data to all the 11 alleles were available, and (ii) 2,665 epitopes used for calculating in silico promiscuity throughout the paper, which were not used for the training of the NetMHCIIpan algorithm. In vitro and predicted promiscuity of the 11 alleles was determined at a 50 nM binding threshold using the selected 216 and 2,665 epitopes, respectively. We used standardized (i.e., z-score) allele promiscuity values for the comparisons. We report a strong correlation between the in silico and in vitro promiscuity scores (Spearman’s rho: 0.78, *P* = 0.004). The red dashed line represents a linear regression line. The underlying data for this figure can be found in S4 Data. IEDB, Immune Epitope Database.
(TIF)

S4 Fig Predicted promiscuity levels are robust to changes in binding threshold, prediction algorithm, and epitope set. The computational prediction of epitope-binding promiscuity was carried out using different binding thresholds: 50 nM for strong binding, which results in the selection of immunodominant peptides [39], and 500 nM and 1,000 nM thresholds, which minimize the number of false negative hits [90]. **(A-C)** The proportion of 2,691 epitopes bound by HLA-DRB1 alleles showed strong correlation between **(A)** 50 nM and 500 nM, **(B)** 50 nM and 1,000 nM, and **(C)** 500 nM and 1,000 nM binding thresholds (Spearman’s rho: 0.95, 0.91, and 0.99 respectively, *P* < 2 × 10^−16^ for all analyses). **(D)** To further ensure the robustness of the predicted promiscuity values, we repeated the predictions using an alternative algorithm, MHC2SKPan as implemented by the MHC2SKPan server [91] (again, promiscuity was calculated using a 50 nM binding threshold). Promiscuity values calculated based on the two algorithms showed a strong correlation (Spearman’s rho: 0.92, *P* < 2 × 10^−16^). **(E)** The proportion of epitopes bound by HLA-DRB1 molecules was independent of the exact epitope set used for the calculations. Specifically, predictions were carried out using epitopes of only extracellular or only intracellular pathogens, and strong correlation was found between binding promiscuity of intracellular and extracellular epitopes (Spearman’s rho: 0.99, *P* < 2 × 10^−16^). Note that promiscuity values are represented in log scale in panels A–C. Red dashed lines indicate linear regression lines (regression line of log-transformed values on panels A–C). The underlying data for this figure can be found in S4 Data.
(TIF)

S5 Fig Increased epitope-binding promiscuity results in the recognition of more pathogens. The number of recognized pathogen species (i.e., those with at least one epitope computationally predicted to be bound by the HLA molecule) is plotted against the computationally predicted promiscuity of the HLA-DRB1 allele. The two variables are strongly correlated (Spearman’s rho: 0.99, *P* < 2 × 10^−16^). Red curve indicates smooth curve fitted using cubic smoothing spline method in R (see Methods). Note that promiscuity is represented in log scale. The underlying data for this figure can be found in S4 Data.
(TIF)

S6 Fig Phylogeny of DRB1 alleles indicates rapid and frequent changes in promiscuity. Phylogeny of DRB1 alleles from Yasukochi and colleagues [47], with predicted epitope-binding promiscuity shown color coded. Alleles were sorted into five equally sized bins according to their promiscuity values. High promiscuity alleles (dark red) are scattered on the tree, suggesting multiple independent origins. Bootstrap percentage values greater than 80% are shown on the internal nodes. The underlying data for this figure can be found in S4 Data.
(TIF)

S7 Fig Amino acid positions in the epitope-binding region of HLA-DRB1 that have a larger impact on promiscuity are more diverse in human populations. Average nonsynonymous nucleotide diversity of an amino acid site in the epitope-binding region (measured as π_A_, see Methods) correlates positively with the site’s promiscuity fragility, measured as the median predicted promiscuity fold difference caused by a random amino acid change at the given site (Spearman’s rho = 0.59, *P* = 0.0075). π_A_ values were calculated for each human population using data on allele frequencies (see Methods). Points represent the average π_A_ values of an amino acid site across all populations. Error bars show standard error. The underlying data for this figure can be found in S4 Data.
(TIF)

S8 Fig Promiscuity fragility and ω values of amino acid sites in the epitope-binding region of HLA-DRB1 correlate positively. ω or dN/dS value (a common measure of positive selection) of an amino acid site in the epitope-binding region of HLA-DRB1 (calculated by [48]) correlates positively with the site’s promiscuity fragility, measured as the median predicted promiscuity fold difference caused by a random amino acid change at the given site (Spearman’s rho: 0.72, *P* = 0.0016, *n* = 16) (see Methods). The underlying data for this figure can be found in S4 Data.
(TIF)

S9 Fig Relationship between intracellular pathogen diversity and HLA class I promiscuity level. Promiscuity scores were calculated based on standardized (i.e., z-score) allele promiscuity values and were averaged in each population group (see Methods). **(A)** A significant positive correlation was found between HLA-A population promiscuity level and intracellular pathogen diversity (Spearman’s rho: 0.52, *P* = 0.003), but not between **(B)** HLA-A population promiscuity level and extracellular pathogen diversity (Spearman’s rho: 0.03, *P* = 0.88). There was a marginally significant positive correlation between **(C)** HLA-B population promiscuity level and intracellular pathogen diversity (Spearman’s rho: 0.34, *P* = 0.03) and **(D)** no significant correlation between extracellular pathogen diversity and HLA-B population promiscuity level (Spearman’s rho: 0.25, *P* = 0.12). One might speculate that there might be no selection for elevated HLA-B promiscuity level due to a dominant balancing selection on this locus (see S1 Text) [7, 8]. Similarly, **(E)** the promiscuity level of HLA-C molecules showed no significant correlation with intracellular pathogen diversity (Spearman’s rho: −0.14, *P* = 0.44). Finally, **(F)** HLA-C promiscuity level showed a marginally significant positive correlation with extracellular pathogen diversity (Spearman’s rho: 0.35, *P* = 0.04). This is surprising, but this preliminary result needs to be considered with caution, and studied further in future works. For detailed explanation of these results, see S1 Text. Population groups were created using the 15th percentile genetic distance cutoff (see Methods). For a list of populations assigned to each group, see S6 Data. For results of multivariate models and obtained upon using alternative distance cutoff values, see S3 Data. Red curves indicate smooth curves fitted using cubic smoothing spline method in R (see Methods). The underlying data for this figure can be found in S4 Data.
(TIF)

S1 Table List of populations and their mean HLA-DRB1 allele promiscuity. (DOCX)

S2 Table The relationship between promiscuity and pathogen diversity is independent of HLA diversity and country size. (DOCX)

S3 Table Results of HLA association studies suggest a protective role of high allele promiscuity in infectious diseases. (DOCX)

S1 Text Pathogen diversity and promiscuity of HLA class I alleles. (DOCX)

S1 Data Epitope sequences of pathogenic species used for allele promiscuity prediction. (XLSX)

S2 Data Normalized promiscuity level of HLA-A, -B, -C, and -DRB1 alleles. (XLSX)

S3 Data Summary of correlation analyses and multivariate models for HLA-I and HLA-II loci. (XLSX)

S4 Data Underlying data for figures. (XLSX)

S5 Data Results of systematic literature search for associations between HLA-DRB1*03 and *12 allelic groups and infectious diseases. (XLSX)

S6 Data List of populations included in the analysis of HLA-A, -B, -C loci. (XLSX)

## Abbreviations

AFND: Allele Frequency Net Database
GIDEON: Global Infectious Diseases and Epidemiology Network
HIV: human immunodeficiency virus
IEDB: Immune Epitope Database
IHIW: International HLA and Immunogenetics Workshop
IHWG: International Histocompatibility Working Group
MHC: major histocompatibility complex
NCBI: National Center for Biotechnology Information
SIV: simian immunodeficiency virus

## References

[1] Neefjes J, Jongsma ML, Paul P, Bakke O. Towards a systems understanding of MHC class I and MHC class II antigen presentation. Nat Rev Immunol. 2011;11(12):823–36. 10.1038/nri3084 22076556

[2] Trowsdale J. The MHC, disease and selection. Immunol Lett. 2011;137(1–2):1–8. 10.1016/j.imlet.2011.01.002 21262263

[3] Spurgin LG, Richardson DS. How pathogens drive genetic diversity: MHC, mechanisms and misunderstandings. Proc Biol Sci. 2010;277(1684):979–88. 10.1098/rspb.2009.2084 20071384 PMC2842774

[4] Sommer S. The importance of immune gene variability (MHC) in evolutionary ecology and conservation. Front Zool. 2005;2:16 10.1186/1742-9994-2-16 16242022 PMC1282567

[5] Barreiro LB, Quintana-Murci L. From evolutionary genetics to human immunology: how selection shapes host defence genes. Nat Rev Genet. 2010;11(1):17–30. 10.1038/nrg2698 19953080

[6] Lenz TL. Adaptive value of novel MHC immune gene variants. Proc Natl Acad Sci U S A. 2018;115(7):1414–6. 10.1073/pnas.1722600115 29386382 PMC5816220

[7] Prugnolle F, Manica A, Charpentier M, Guegan JF, Guernier V, Balloux F. Pathogen-driven selection and worldwide HLA class I diversity. Curr Biol. 2005;15(11):1022–7. 10.1016/j.cub.2005.04.050 15936272

[8] Sanchez-Mazas A, Lemaitre JF, Currat M. Distinct evolutionary strategies of human leucocyte antigen loci in pathogen-rich environments. Philos Trans R Soc Lond B Biol Sci. 2012;367(1590):830–9. 10.1098/rstb.2011.0312 22312050 PMC3267122

[9] Chappell P, Meziane el K, Harrison M, Magiera L, Hermann C, Mears L, et al Expression levels of MHC class I molecules are inversely correlated with promiscuity of peptide binding. Elife. 2015;4:e05345 10.7554/eLife.05345 25860507 PMC4420994

[10] Kaufman J. Generalists and Specialists: A New View of How MHC Class I Molecules Fight Infectious Pathogens. Trends Immunol. 2018;39(5):367–79. 10.1016/j.it.2018.01.001 29396014 PMC5929564

[11] Paul S, Weiskopf D, Angelo MA, Sidney J, Peters B, Sette A. HLA class I alleles are associated with peptide-binding repertoires of different size, affinity, and immunogenicity. J Immunol. 2013;191(12):5831–9. 10.4049/jimmunol.1302101 24190657 PMC3872965

[12] Kosmrlj A, Read EL, Qi Y, Allen TM, Altfeld M, Deeks SG, et al Effects of thymic selection of the T-cell repertoire on HLA class I-associated control of HIV infection. Nature. 2010;465(7296):350–4. 10.1038/nature08997 20445539 PMC3098720

[13] Mothe BR, Southwood S, Sidney J, English AM, Wriston A, Hoof I, et al Peptide-binding motifs associated with MHC molecules common in Chinese rhesus macaques are analogous to those of human HLA supertypes and include HLA-B27-like alleles. Immunogenetics. 2013;65(5):371–86. 10.1007/s00251-013-0686-9 23417323 PMC3633659

[14] Boonyanuwat K, Thummabutra S, Sookmanee N, Vatchavalkhu V, Siripholvat V. Influences of major histocompatibility complex class I haplotypes on avian influenza virus disease traits in Thai indigenous chickens. Animal science journal. 2006;77(3):285–9.

[15] Simonsen M. The MHC of the chicken, genomic structure, gene products, and resistance to oncogenic DNA and RNA viruses. Veterinary immunology and immunopathology. 1987;17(1–4):243–53. 2829414 10.1016/0165-2427(87)90144-9

[16] Banat GR, Tkalcic S, Dzielawa JA, Jackwood MW, Saggese MD, Yates L, et al Association of the chicken MHC B haplotypes with resistance to avian coronavirus. Developmental & Comparative Immunology. 2013;39(4):430–7. 23178407 10.1016/j.dci.2012.10.006 PMC7103219

[17] McBride R, Cutting J, Schierman L, Strebel F, Watanabe D. MHC gene control of growth of avian sarcoma virus‐induced tumours in chickens: a study on the role of virus strain. International Journal of Immunogenetics. 1981;8(3):207–14. 10.1111/j.1744-313x.1981.tb00758.x 6267139

[18] Karlsson EK, Kwiatkowski DP, Sabeti PC. Natural selection and infectious disease in human populations. Nat Rev Genet. 2014;15(6):379–93. 10.1038/nrg3734 24776769 PMC4912034

[19] Robinson J, Halliwell JA, Hayhurst JD, Flicek P, Parham P, Marsh SG. The IPD and IMGT/HLA database: allele variant databases. Nucleic Acids Res. 2015;43(Database issue):D423–31. 10.1093/nar/gku1161 25414341 PMC4383959

[20] Yasukochi Y, Satta Y. Current perspectives on the intensity of natural selection of MHC loci. Immunogenetics. 2013;65(6):479–83. 10.1007/s00251-013-0693-x 23549729 PMC3651823

[21] Pierini F, Lenz TL. Divergent allele advantage at human MHC genes: signatures of past and ongoing selection. Mol Biol Evol. 2018. 10.1093/molbev/msy116 PMC6106954 29893875

[22] Yasukochi Y, Satta Y. A human-specific allelic group of the MHC DRB1 gene in primates. J Physiol Anthropol. 2014;33:14 10.1186/1880-6805-33-14 24928070 PMC4072476

[23] Quintana-Murci L. Understanding rare and common diseases in the context of human evolution. Genome Biol. 2016;17(1):225 10.1186/s13059-016-1093-y 27821149 PMC5098287

[24] Satta Y O 'HUigin C, Takahata N, Klein J. Intensity of natural selection at the major histocompatibility complex loci. Proc Natl Acad Sci U S A. 1994;91(15):7184–8. 8041766 10.1073/pnas.91.15.7184 PMC44363

[25] Miyadera H, Ohashi J, Lernmark A, Kitamura T, Tokunaga K. Cell-surface MHC density profiling reveals instability of autoimmunity-associated HLA. J Clin Invest. 2015;125(1):275–91. 10.1172/JCI74961 25485681 PMC4382229

[26] Manczinger M, Kemeny L.. Peptide presentation by HLA-DQ molecules is associated with the development of immune tolerance. PeerJ 2018;6:e5118 10.7717/peerj.5118 30002966 PMC6034589

[27] van Lith M, McEwen-Smith RM, Benham AM. HLA-DP, HLA-DQ, and HLA-DR have different requirements for invariant chain and HLA-DM. J Biol Chem. 2010;285(52):40800–8. 10.1074/jbc.M110.148155 20959457 PMC3003381

[28] Chowell D, Morris LGT, Grigg CM, Weber JK, Samstein RM, Makarov V, et al Patient HLA class I genotype influences cancer response to checkpoint blockade immunotherapy. Science. 2018;359(6375):582–7. 10.1126/science.aao4572 29217585 PMC6057471

[29] Anczurowski M, Hirano N. Mechanisms of HLA-DP Antigen Processing and Presentation Revisited. Trends Immunol. 2018. 10.1016/j.it.2018.10.008 30416081

[30] Karosiene E, Rasmussen M, Blicher T, Lund O, Buus S, Nielsen M. NetMHCIIpan-3.0, a common pan-specific MHC class II prediction method including all three human MHC class II isotypes, HLA-DR, HLA-DP and HLA-DQ. Immunogenetics. 2013;65(10):711–24. 10.1007/s00251-013-0720-y 23900783 PMC3809066

[31] Andreatta M, Trolle T, Yan Z, Greenbaum JA, Peters B, Nielsen M. An automated benchmarking platform for MHC class II binding prediction methods. Bioinformatics. 2018;34(9):1522–8. 10.1093/bioinformatics/btx820 29281002 PMC5925780

[32] Fernandez-Vina MA, Klein JP, Haagenson M, Spellman SR, Anasetti C, Noreen H, et al Multiple mismatches at the low expression HLA loci DP, DQ, and DRB3/4/5 associate with adverse outcomes in hematopoietic stem cell transplantation. Blood. 2013;121(22):4603–10. 10.1182/blood-2013-02-481945 23596045 PMC3668493

[33] Boegel S, Lower M, Bukur T, Sorn P, Castle JC, Sahin U. HLA and proteasome expression body map. BMC Med Genomics. 2018;11(1):36 10.1186/s12920-018-0354-x 29587858 PMC5872580

[34] Hofer TP, Frankenberger M, Heimbeck I, Burggraf D, Wjst M, Wright AK, et al Decreased expression of HLA-DQ and HLA-DR on cells of the monocytic lineage in cystic fibrosis. J Mol Med (Berl). 2014;92(12):1293–304. 25146850 10.1007/s00109-014-1200-z

[35] Gonzalez-Galarza FF, Takeshita LY, Santos EJ, Kempson F, Maia MH, da Silva AL, et al Allele frequency net 2015 update: new features for HLA epitopes, KIR and disease and HLA adverse drug reaction associations. Nucleic Acids Res. 2015;43(Database issue):D784–8. 10.1093/nar/gku1166 25414323 PMC4383964

[36] Meyer D, Singe R, Mack S, Lancaster A, Nelson M, Erlich H, et al. Immunobiology of the Human MHC: Proceedings of the 13th International Histocompatibility Workshop and Conference. Seattle, WA: IHWG Press; 2007.

[37] Riccio ME, Buhler S, Nunes JM, Vangenot C, Cuenod M, Currat M, et al 16(th) IHIW: analysis of HLA population data, with updated results for 1996 to 2012 workshop data (AHPD project report). Int J Immunogenet. 2013;40(1):21–30. 10.1111/iji.12033 23280239

[38] Vita R, Overton JA, Greenbaum JA, Ponomarenko J, Clark JD, Cantrell JR, et al The immune epitope database (IEDB) 3.0. Nucleic Acids Res. 2015;43(Database issue):D405–12. 10.1093/nar/gku938 25300482 PMC4384014

[39] Oyarzun P, Ellis JJ, Boden M, Kobe B. PREDIVAC: CD4+ T-cell epitope prediction for vaccine design that covers 95% of HLA class II DR protein diversity. BMC Bioinformatics. 2013;14:52 10.1186/1471-2105-14-52 23409948 PMC3598884

[40] Coker RJ, Hunter BM, Rudge JW, Liverani M, Hanvoravongchai P. Emerging infectious diseases in southeast Asia: regional challenges to control. Lancet. 2011;377(9765):599–609. 10.1016/S0140-6736(10)62004-1 21269678 PMC7159088

[41] Edberg SC. Global Infectious Diseases and Epidemiology Network (GIDEON): a world wide Web-based program for diagnosis and informatics in infectious diseases. Clin Infect Dis. 2005;40(1):123–6. 10.1086/426549 15614701

[42] Fumagalli M, Pozzoli U, Cagliani R, Comi GP, Riva S, Clerici M, et al Parasites represent a major selective force for interleukin genes and shape the genetic predisposition to autoimmune conditions. J Exp Med. 2009;206(6):1395–408. 10.1084/jem.20082779 19468064 PMC2715056

[43] Sun H, Yang Z, Lin K, Liu S, Huang K, Wang X, et al The Adaptive Change of HLA-DRB1 Allele Frequencies Caused by Natural Selection in a Mongolian Population That Migrated to the South of China. PLoS ONE. 2015;10(7):e0134334 10.1371/journal.pone.0134334 26230582 PMC4521750

[44] Crump JA, Luby SP, Mintz ED. The global burden of typhoid fever. Bull World Health Organ. 2004;82(5):346–53. 15298225 PMC2622843

[45] Okamoto K, Brown JD. Hepatosplenic schistosomiasis presenting as spontaneous hemoperitoneum in a Filipino immigrant. Am J Med Sci. 2013;346(4):334–7. 10.1097/MAJ.0b013e31828f4bee 23588267

[46] Nair N, Wares F, Sahu S. Tuberculosis in the WHO South-East Asia Region. Bull World Health Organ. 2010;88(3):164 10.2471/BLT.09.073874 20428378 PMC2828794

[47] Yasukochi Y, Satta Y. Nonsynonymous substitution rate heterogeneity in the peptide-binding region among different HLA-DRB1 lineages in humans. G3 (Bethesda). 2014;4(7):1217–26. 24793785 10.1534/g3.114.011726 PMC4455771

[48] Furlong RF, Yang Z. Diversifying and purifying selection in the peptide binding region of DRB in mammals. J Mol Evol. 2008;66(4):384–94. 10.1007/s00239-008-9092-6 18347751

[49] Karnes JH, Bastarache L, Shaffer CM, Gaudieri S, Xu Y, Glazer AM, et al Phenome-wide scanning identifies multiple diseases and disease severity phenotypes associated with HLA variants. Science Translational Medicine. 2017;9(389). 10.1126/scitranslmed.aai8708 PMC5563969 28490672

[50] Rossman MD, Thompson B, Frederick M, Maliarik M, Iannuzzi MC, Rybicki BA, et al HLA-DRB1*1101: a significant risk factor for sarcoidosis in blacks and whites. Am J Hum Genet. 2003;73(4):720–35. 10.1086/378097 14508706 PMC1180597

[51] Hewitt EW. The MHC class I antigen presentation pathway: strategies for viral immune evasion. Immunology. 2003;110(2):163–9. 10.1046/j.1365-2567.2003.01738.x 14511229 PMC1783040

[52] Trowsdale J, Knight JC. Major histocompatibility complex genomics and human disease. Annu Rev Genomics Hum Genet. 2013;14:301–23. 10.1146/annurev-genom-091212-153455 23875801 PMC4426292

[53] Lenz TL, Spirin V, Jordan DM, Sunyaev SR. Excess of Deleterious Mutations around HLA Genes Reveals Evolutionary Cost of Balancing Selection. Mol Biol Evol. 2016;33(10):2555–64. 10.1093/molbev/msw127 27436009 PMC5026253

[54] Ejsmond MJ, Radwan J. Red Queen Processes Drive Positive Selection on Major Histocompatibility Complex (MHC) Genes. PLoS Comput Biol. 2015;11(11):e1004627 10.1371/journal.pcbi.1004627 26599213 PMC4658181

[55] Crawford A, Macleod M, Schumacher T, Corlett L, Gray D. Primary T cell expansion and differentiation in vivo requires antigen presentation by B cells. J Immunol. 2006;176(6):3498–506. 16517718 10.4049/jimmunol.176.6.3498

[56] Thursz MR, Kwiatkowski D, Allsopp CE, Greenwood BM, Thomas HC, Hill AV. Association between an MHC class II allele and clearance of hepatitis B virus in the Gambia. N Engl J Med. 1995;332(16):1065–9. 10.1056/NEJM199504203321604 7898524

[57] Paludan C, Schmid D, Landthaler M, Vockerodt M, Kube D, Tuschl T, et al Endogenous MHC class II processing of a viral nuclear antigen after autophagy. Science. 2005;307(5709):593–6. 10.1126/science.1104904 15591165

[58] Sveinbjornsson G, Gudbjartsson DF, Halldorsson BV, Kristinsson KG, Gottfredsson M, Barrett JC, et al HLA class II sequence variants influence tuberculosis risk in populations of European ancestry. Nature genetics. 2016;48(3):318–22. 10.1038/ng.3498 26829749 PMC5081101

[59] Moutsianas L, Jostins L, Beecham AH, Dilthey AT, Xifara DK, Ban M, et al Class II HLA interactions modulate genetic risk for multiple sclerosis. Nature genetics. 2015;47(10):1107–13. 10.1038/ng.3395 26343388 PMC4874245

[60] Okada Y, Momozawa Y, Ashikawa K, Kanai M, Matsuda K, Kamatani Y, et al Construction of a population-specific HLA imputation reference panel and its application to Graves' disease risk in Japanese. Nature genetics. 2015;47(7):798–802. 10.1038/ng.3310 26029868

[61] Lenz TL, Deutsch AJ, Han B, Hu X, Okada Y, Eyre S, et al Widespread non-additive and interaction effects within HLA loci modulate the risk of autoimmune diseases. Nat Genet. 2015;47(9):1085–90. 10.1038/ng.3379 26258845 PMC4552599

[62] McLaren PJ, Coulonges C, Bartha I, Lenz TL, Deutsch AJ, Bashirova A, et al Polymorphisms of large effect explain the majority of the host genetic contribution to variation of HIV-1 virus load. Proc Natl Acad Sci U S A. 2015;112(47):14658–63. 10.1073/pnas.1514867112 26553974 PMC4664299

[63] Leinders-Zufall T, Brennan P, Widmayer P, S PC, Maul-Pavicic A, Jager M, et al MHC class I peptides as chemosensory signals in the vomeronasal organ. Science. 2004;306(5698):1033–7. 10.1126/science.1102818 15528444

[64] Woelfing B, Traulsen A, Milinski M, Boehm T. Does intra-individual major histocompatibility complex diversity keep a golden mean? Philos Trans R Soc Lond B Biol Sci. 2009;364(1513):117–28. 10.1098/rstb.2008.0174 18926972 PMC2666699

[65] Jiang W, Boder ET. High-throughput engineering and analysis of peptide binding to class II MHC. Proc Natl Acad Sci U S A. 2010;107(30):13258–63. 10.1073/pnas.1006344107 20622157 PMC2922119

[66] Taneja V, David CS. HLA class II transgenic mice as models of human diseases. Immunol Rev. 1999;169:67–79. 10450509 10.1111/j.1600-065x.1999.tb01307.x

[67] Yucesoy B, Talzhanov Y, Johnson VJ, Wilson NW, Biagini RE, Wang W, et al Genetic variants within the MHC region are associated with immune responsiveness to childhood vaccinations. Vaccine. 2013;31(46):5381–91. 10.1016/j.vaccine.2013.09.026 24075919 PMC4640212

[68] Liu Z, Colovai AI, Tugulea S, Reed EF, Fisher PE, Mancini D, et al Indirect recognition of donor HLA-DR peptides in organ allograft rejection. J Clin Invest. 1996;98(5):1150–7. 10.1172/JCI118898 8787678 PMC507537

[69] Marty R, Kaabinejadian S, Rossell D, Slifker MJ, van de Haar J, Engin HB, et al MHC-I Genotype Restricts the Oncogenic Mutational Landscape. Cell. 2017;171(6):1272–83 e15. 10.1016/j.cell.2017.09.050 29107334 PMC5711564

[70] Marty R, Thompson WK, Salem RM, Zanetti M, Carter H. Evolutionary Pressure against MHC Class II Binding Cancer Mutations. Cell. 2018;175(2):416–28 e13. 10.1016/j.cell.2018.08.048 30245014 PMC6482006

[71] UniProt Consortium T. UniProt: the universal protein knowledgebase. Nucleic Acids Res. 2018;46(5):2699 10.1093/nar/gky092 29425356 PMC5861450

[72] Sievers F, Wilm A, Dineen D, Gibson TJ, Karplus K, Li W, et al Fast, scalable generation of high‐quality protein multiple sequence alignments using Clustal Omega. Molecular Systems Biology. 2011;7(1). 10.1038/msb.2011.75 PMC3261699 21988835

[73] Yang K, Zhang L. Performance comparison between k-tuple distance and four model-based distances in phylogenetic tree reconstruction. Nucleic Acids Res. 2008;36(5):e33 10.1093/nar/gkn075 18296485 PMC2275138

[74] Jurtz V, Paul S, Andreatta M, Marcatili P, Peters B, Nielsen M. NetMHCpan-4.0: Improved Peptide-MHC Class I Interaction Predictions Integrating Eluted Ligand and Peptide Binding Affinity Data. J Immunol. 2017;199(9):3360–8. 10.4049/jimmunol.1700893 28978689 PMC5679736

[75] Rogers JS. Deriving phylogenetic trees from allele frequencies: a comparison of nine genetic distances. Systematic Biology. 1986;35(3):297–310.

[76] Rosenberg NA, Pritchard JK, Weber JL, Cann HM, Kidd KK, Zhivotovsky LA, et al Genetic structure of human populations. Science. 2002;298(5602):2381–5. 10.1126/science.1078311 12493913

[77] Kumar S, Stecher G, Tamura K. MEGA7: Molecular Evolutionary Genetics Analysis Version 7.0 for Bigger Datasets. Mol Biol Evol. 2016;33(7):1870–4. 10.1093/molbev/msw054 27004904 PMC8210823

[78] Hughes AL, Nei M. Nucleotide substitution at major histocompatibility complex class II loci: evidence for overdominant selection. Proc Natl Acad Sci U S A. 1989;86(3):958–62. 2492668 10.1073/pnas.86.3.958 PMC286598

[79] Hughes AL, Yeager M. Natural selection at major histocompatibility complex loci of vertebrates. Annual review of genetics. 1998;32(1):415–35. 10.1146/annurev.genet.32.1.415 9928486

[80] Yasukochi Y, Kurosaki T, Yoneda M, Koike H, Satta Y. MHC class II DQB diversity in the Japanese black bear, Ursus thibetanus japonicus. BMC Evol Biol. 2012;12:230 10.1186/1471-2148-12-230 23190438 PMC3575356

[81] Sawyer S. Statistical tests for detecting gene conversion. Mol Biol Evol. 1989;6(5):526–38. 10.1093/oxfordjournals.molbev.a040567 2677599

[82] Martin D, Rybicki E. RDP: detection of recombination amongst aligned sequences. Bioinformatics. 2000;16(6):562–3. 10980155 10.1093/bioinformatics/16.6.562

[83] Martin DP, Murrell B, Golden M, Khoosal A, Muhire B. RDP4: Detection and analysis of recombination patterns in virus genomes. Virus Evol. 2015;1(1):vev003 10.1093/ve/vev003 27774277 PMC5014473

[84] Grant BJ, Rodrigues AP, ElSawy KM, McCammon JA, Caves LS. Bio3d: an R package for the comparative analysis of protein structures. Bioinformatics. 2006;22(21):2695–6. 10.1093/bioinformatics/btl461 16940322

[85] Nei M, Li WH. Mathematical model for studying genetic variation in terms of restriction endonucleases. Proc Natl Acad Sci U S A. 1979;76(10):5269–73. 291943 10.1073/pnas.76.10.5269 PMC413122

[86] Hughes AL, Friedman R, Rivailler P, French JO. Synonymous and nonsynonymous polymorphisms versus divergences in bacterial genomes. Mol Biol Evol. 2008;25(10):2199–209. 10.1093/molbev/msn166 18667439 PMC2734133

[87] Librado P, Rozas J. DnaSP v5: a software for comprehensive analysis of DNA polymorphism data. Bioinformatics. 2009;25(11):1451–2. 10.1093/bioinformatics/btp187 19346325

[88] Ihaka R, Gentleman R. R: a language for data analysis and graphics. Journal of computational and graphical statistics. 1996;5(3):299–314.

[89] De Boor C. A practical guide to splines: Springer-Verlag New York; 1978.

[90] Greenbaum J, Sidney J, Chung J, Brander C, Peters B, Sette A. Functional classification of class II human leukocyte antigen (HLA) molecules reveals seven different supertypes and a surprising degree of repertoire sharing across supertypes. Immunogenetics. 2011;63(6):325–35. 10.1007/s00251-011-0513-0 21305276 PMC3626422

[91] Guo L, Luo C, Zhu S. MHC2SKpan: a novel kernel based approach for pan-specific MHC class II peptide binding prediction. BMC Genomics. 2013;14 Suppl 5:S11. 10.1186/1471-2164-14-S5-S11 PMC3852073 24564280

