# Supplementary figures and images for: Pathogen diversity drives the evolution of generalist MHC-II alleles in human populations
Source: PLoS Biol. 2019 Jan 31;17(1):e3000131. doi: 10.1371/journal.pbio.3000131 (PMC6372212; doi:10.1371/journal.pbio.3000131)

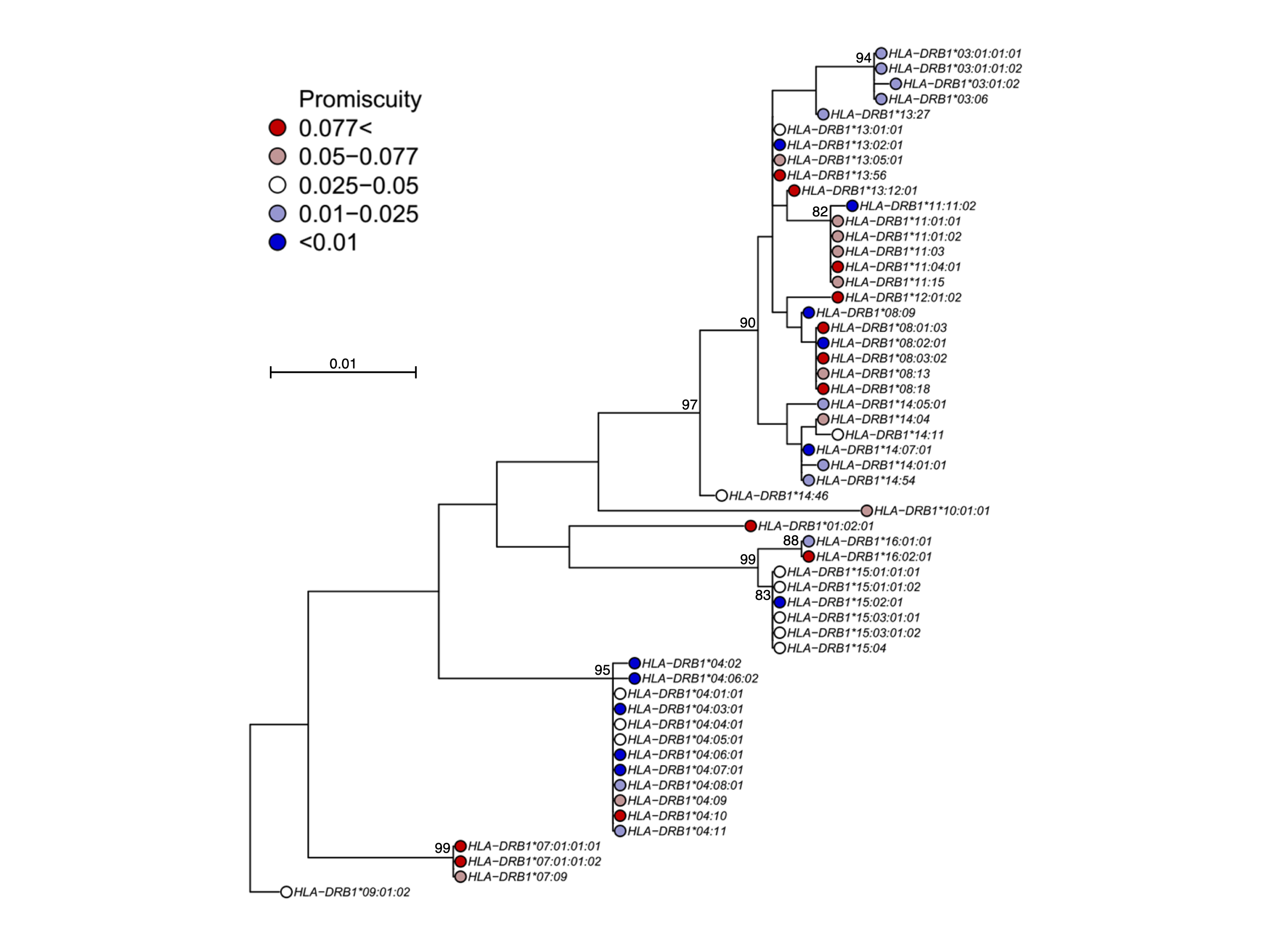

Supplement: S6 Fig — Phylogeny of DRB1 alleles from Yasukochi and colleagues [47], with predicted epitope-binding promiscuity shown color coded. Alleles were sorted into five equally sized bins according to their promiscuity values. High promiscuity alleles (dark red) are scattered on the tree, suggesting multiple independent origins. Bootstrap percentage values greater than 80% are shown on the internal nodes. The underlying data for this figure can be found in S4 Data. (TIF) [file pbio.3000131.s006.tif]

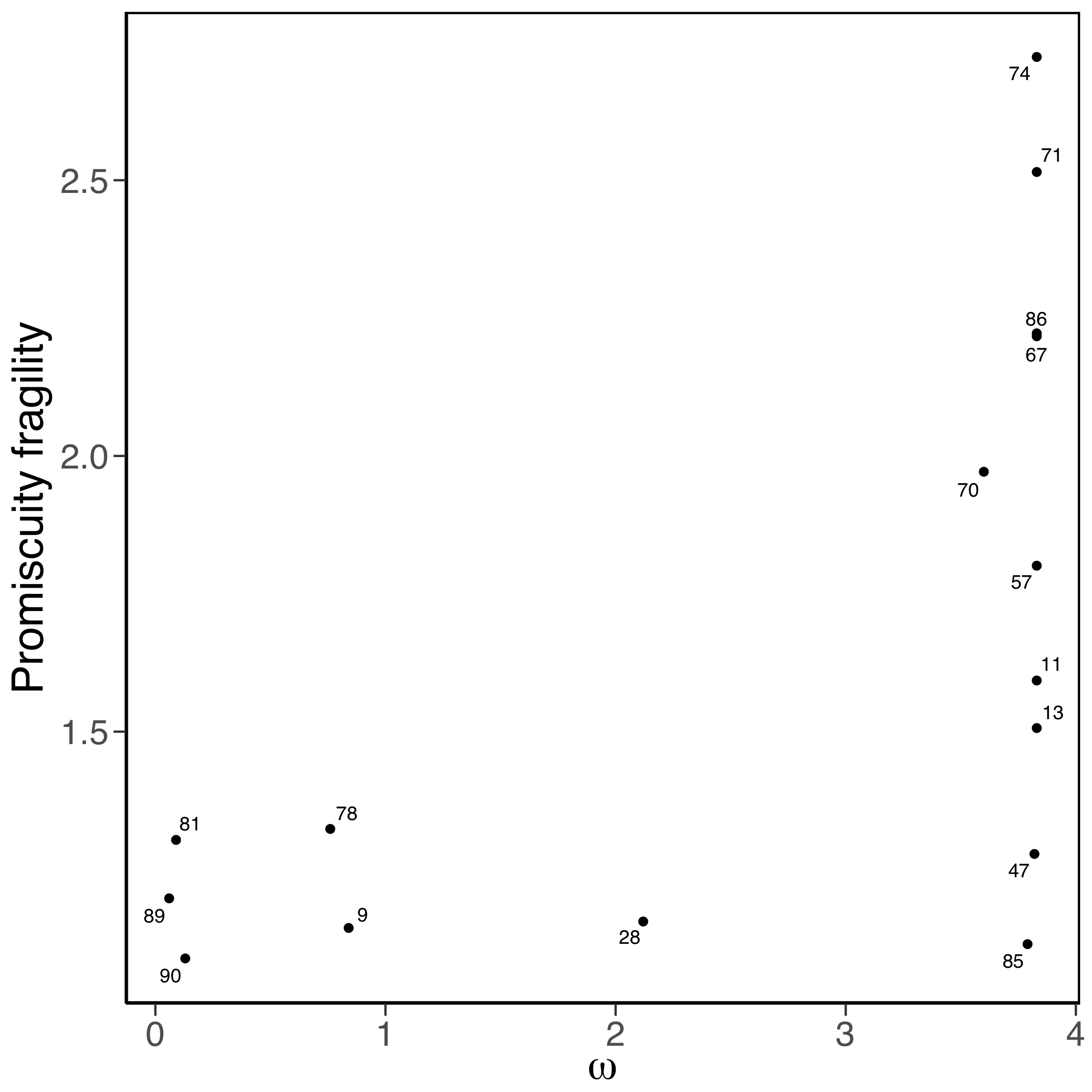

Supplement: S8 Fig — ω or dN/dS value (a common measure of positive selection) of an amino acid site in the epitope-binding region of HLA-DRB1 (calculated by [48]) correlates positively with the site’s promiscuity fragility, measured as the median predicted promiscuity fold difference caused by a random amino acid change at the given site (Spearman’s rho: 0.72, P = 0.0016, n = 16) (see Methods). The underlying data for this figure can be found in S4 Data. (TIF) [file pbio.3000131.s008.tif]
