# Supplementary material for: Pathogen diversity drives the evolution of generalist MHC-II alleles in human populations
Source: PLoS Biol. 2019 Jan 31;17(1):e3000131. doi: 10.1371/journal.pbio.3000131 (PMC6372212; doi:10.1371/journal.pbio.3000131)
Supplement: S1 Table — (DOCX) [file pbio.3000131.s010.docx]

**S1 Table. List of populations and their mean HLA-DRB1 allele promiscuity.** The first four columns show population groups when using different genetic distance cutoff values for classification (see Methods). Standardized (i.e. z-score) in silico and in vitro mean population promiscuity scores are shown. Abbreviations: AFND: Allele Frequencies Net Database, NA: insufficient information on in vitro allele promiscuity.

| **Pop. Groups  (cutoff in percentile)** | | | | **Population** | **Country** | **Source** | **Predicted**  **promiscuity** | **In vitro**  **promiscuity** | **Most frequent allele** | | | **Information supporting that population is native** |
| --- | --- | --- | --- | --- | --- | --- | --- | --- | --- | --- | --- | --- |
| **1%** | **5%** | **10%** | **15%** |  |  |  |  |  | **Name** | **Prevalence** | **Predicted promiscuity** |  |
| 56 | 11 | 3 | 1 | Guarani-Kaiowa | Brazil | dbMHC | -0.30 | 1.11 | 1602 | 0.48 | -0.12 | [1, 2] |
| 55 | 11 | 3 | 1 | Guarani-Nandewa | Brazil | dbMHC | -0.09 | NA | 1602 | 0.27 | -0.12 | [1, 2] |
| 6 | 1 | 2 | 2 | Hakka | Taiwan | dbMHC | -0.23 | 0.08 | 0901 | 0.16 | -0.51 | [1, 2] |
| 5 | 2 | 2 | 2 | Japan Central | Japan | AFND | -0.22 | 0.17 | 0901 | 0.15 | -0.51 | [2, 3] |
| 5 | 15 | 4 | 2 | Japan Kyoto and Osaka | Japan | AFND | -0.24 | 0.26 | 0405 0901 | 0.17 | -0.47 -0.51 | [2, 4] |
| 2 | 2 | 2 | 2 | Korean 200 | South Korea | dbMHC | -0.14 | 0.14 | 0901 | 0.17 | -0.51 | [1, 2] |
| 6 | 1 | 4 | 2 | Minnan | Taiwan | dbMHC | -0.13 | 0.27 | 0901 | 0.17 | -0.51 | [1, 2] |
| 2 | 2 | 2 | 2 | South Korea pop 11 | South Korea | AFND | -0.11 | 0.17 | 0901 | 0.18 | -0.51 | [2, 5] |
| 2 | 2 | 2 | 2 | South Korea pop 2 | South Korea | AFND | -0.13 | 0.14 | 0405 1302 | 0.35 | -0.47 -0.75 | [2, 6] |
| 2 | 2 | 2 | 2 | South Korea pop 3 | South Korea | AFND | -0.18 | 0.11 | 0405 0901 | 0.39 | -0.47 -0.51 | [2, 7] |
| 2 | 2 | 2 | 2 | South Korea pop 4 | South Korea | AFND | -0.17 | 0.16 | 1302 | 0.12 | -0.75 | [2, 8] |
| 2 | 2 | 2 | 2 | South Korea pop 6 | South Korea | AFND | -0.17 | 0.12 | 0901 | 0.15 | -0.51 | [2, 9] |
| 6 | 1 | 4 | 2 | Taiwan Han Chinese | Taiwan | AFND | -0.17 | 0.17 | 0901 | 0.29 | -0.51 | [2] |
| 6 | 1 | 4 | 2 | Taiwan Minnan and Hakka | Taiwan | AFND | -0.15 | 0.22 | 0901 | 0.17 | -0.51 | [10] |
| 1 | 9 | 2 | 2 | Tuva | Russia | dbMHC | -0.15 | -0.08 | 1501 | 0.16 | -0.49 | [1, 2] |
| 74 | 5 | 6 | 3 | 16WS-15 Russians-Vologda | Russia | [11] | -0.08 | -0.04 | 0701 | 0.19 | -0.15 | [2, 11] |
| 3 | 5 | 6 | 3 | 16WS-27 British-Wales | UK | [11] | -0.22 | -0.08 | 0701 | 0.17 | -0.15 | [2, 11] |
| 3 | 5 | 6 | 3 | England North West | UK | AFND | -0.24 | -0.08 | 1501 | 0.15 | -0.49 | [12] AFND |
| 57 | 41 | 38 | 3 | Finn 90 | Finland | dbMHC | -0.18 | -0.13 | 1301 | 0.15 | -0.6 | [1, 2] |
| 3 | 5 | 6 | 3 | Irish | Ireland | dbMHC | -0.22 | -0.06 | 1501 | 0.21 | -0.49 | [1, 2] |
| 33 | 8 | 5 | 3 | Netherlands | Netherlands | AFND | -0.21 | -0.18 | 0301 | 0.45 | -0.66 | [2] |
| 3 | 5 | 6 | 3 | Norway | Norway | AFND | -0.28 | -0.12 | 1501 | 0.16 | -0.49 | [13] |
| 7 | 5 | 5 | 3 | Russia Northwest Slavic | Russia | AFND | -0.10 | -0.15 | 0701 | 0.19 | -0.15 | [14] |
| 18 | 9 | 6 | 3 | Sweden Southern Sami | Sweden | AFND | -0.28 | -0.06 | 1501 | 0.30 | -0.49 | [15] |
| 7 | 5 | 5 | 4 | 16WS-36 French-Montpellier | France | [11] | -0.13 | -0.13 | 0701 | 0.35 | -0.15 | [2, 11] |
| 11 | 8 | 5 | 4 | 16WS-37 Croatians | Croatia | [11] | -0.16 | -0.24 | 0301 | 0.34 | -0.66 | [2, 11] |
| 43 | 34 | 9 | 4 | Algerian 99 | Algeria | dbMHC | -0.33 | -0.38 | 0301 | 0.18 | -0.66 | [1, 2] |
| 51 | 5 | 5 | 4 | Czech | Czech Republic | dbMHC | -0.10 | -0.14 | 0701 | 0.12 | -0.15 | [1, 2] |
| 10 | 10 | 9 | 4 | Madeira pop 2 | Portugal | AFND | 0.04 | -0.18 | 0701 | 0.12 | -0.15 | [16] |
| 10 | 8 | 5 | 4 | Madeira | Portugal | AFND | -0.11 | -0.28 | 0701 | 0.15 | -0.15 | [2, 17] |
| 54 | 10 | 9 | 4 | Moroccan 99 | Morocco | dbMHC | -0.20 | -0.39 | 0301 | 0.23 | -0.66 | [1, 2] |
| 1 | 9 | 2 | 4 | Russia Tuva pop 2 | Russia | AFND | -0.15 | -0.08 | 1501 | 0.17 | -0.49 | [18] |
| 16 | 13 | 9 | 4 | Saudi Arabia Guraiat and Hail | Saudi Arabia | AFND | -0.32 | -0.39 | 0701 | 0.15 | -0.15 | AFND |
| 21 | 6 | 8 | 4 | South Africa Black | South Africa | AFND | -0.24 | -0.30 | 1301 | 0.14 | -0.6 | [19] |
| 10 | 10 | 9 | 4 | Spain Murcia | Spain | AFND | 0.00 | -0.23 | 0701 | 0.16 | -0.15 | [20] |
| 12 | 10 | 9 | 4 | Tunisia pop 3 | Tunisia | AFND | -0.18 | -0.54 | 0301 | 0.15 | -0.66 | [21] |
| 73 | 3 | 5 | 4 | Turk | Turkey | dbMHC | -0.20 | -0.26 | 1101 | 0.10 | -0.22 | [1, 2] |
| 4 | 3 | 7 | 5 | 15WS-12 Greeks | Greece | [11] | -0.05 | -0.19 | 1104 | 0.12 | 1.15 | [2, 11] |
| 9 | 3 | 7 | 5 | 15WS-19 Macedonians | Macedonia | [11] | -0.07 | NA | 1601 | 0.75 | -0.69 | [2, 11] |
| 4 | 3 | 7 | 5 | 16WS-44 Greeks | Greece | [11] | -0.05 | -0.21 | 1104 | 0.23 | 1.15 | [2, 11] |
| 75 | 57 | 7 | 5 | 16WS-52 Kosovo-Albanians | Albania | [11] | -0.29 | -0.30 | 1101 | 0.22 | -0.22 | [2, 11] |
| 9 | 3 | 5 | 5 | Macedonia pop 1 | Macedonia | AFND | -0.04 | -0.13 | 1601 | 0.57 | -0.69 | [22] |
| 4 | 3 | 7 | 5 | Macedonia pop 3 | Macedonia | AFND | -0.13 | -0.24 | 1104 | 0.30 | 1.15 | [23] |
| 11 | 8 | 5 | 5 | Slovenian | Slovenia | dbMHC | -0.17 | -0.09 | 0301 | 0.12 | -0.66 | [1, 2] |
| 42 | 13 | 9 | 6 | Moroccan 98 | Morocco | dbMHC | -0.20 | -0.39 | 0701 | 0.29 | -0.15 | [1, 2] |
| 23 | 13 | 9 | 6 | Saudi Arabia pop 5 | Saudi Arabia | AFND | -0.25 | -0.36 | 0701 | 0.32 | -0.15 | [24] |
| 37 | 29 | 27 | 7 | Cameroon | Cameroon | AFND | -0.14 | NA | 1503 | 0.37 | -0.43 | [25] |
| 72 | 56 | 8 | 7 | Rwandan | Rwanda | dbMHC | -0.05 | NA | 1503 | 0.15 | -0.43 | [1, 2] |
| 52 | 6 | 8 | 7 | Shona | Zimbabwe | dbMHC | -0.07 | -0.22 | 1503 | 0.17 | -0.43 | [1, 2] |
| 27 | 24 | 15 | 8 | China Yunnan Hani | China | AFND | 0.18 | 0.94 | 1202 | 0.23 | 1.58 | [26] |
| 28 | 4 | 10 | 8 | China Yunnan Province Lisu | China | AFND | 0.14 | 0.74 | 1202 | 0.23 | 1.58 | [27] |
| 29 | 4 | 10 | 8 | China Yunnan Province Nu | China | AFND | 0.14 | 0.69 | 1202 | 0.32 | 1.58 | [27] |
| 35 | 14 | 11 | 9 | China Wanwei, Wutou and Shanxin Islands Jing | China | AFND | -0.09 | 0.37 | 1202 | 0.15 | 1.58 | [28] |
| 8 | 7 | 1 | 9 | Kinh | Vietnam | dbMHC | 0.22 | 0.50 | 1202 | 0.28 | 1.58 | [1, 2] |
| 48 | 7 | 1 | 9 | Malay | Malaysia | dbMHC | 0.30 | 0.58 | 1202 | 0.32 | 1.58 | [1, 2] |
| 8 | 7 | 1 | 9 | Vietnam Hanoi Kinh pop 2 | Vietnam | AFND | 0.30 | 0.58 | 1202 | 0.17 | 1.58 | [2] |
| 67 | 51 | 13 | 10 | Paiwan 51 | Taiwan | dbMHC | 0.30 | 0.26 | 1202 | 0.20 | 1.58 | [1, 2] |
| 65 | 49 | 13 | 10 | Rukai | Taiwan | dbMHC | 0.42 | 0.50 | 1202 | 0.11 | 1.58 | [1, 2] |
| 24 | 14 | 11 | 11 | China Guangxi Region Maonan | China | AFND | -0.05 | 0.68 | 1202 | 0.16 | 1.58 | [29] |
| 25 | 12 | 11 | 11 | China Southwest Dai | China | AFND | -0.27 | 0.60 | 1602 | 0.28 | -0.12 | [30] |
| 50 | 12 | 11 | 11 | Muong | Vietnam | dbMHC | -0.21 | 0.53 | 1602 | 0.12 | -0.12 | [1, 2] |
| 66 | 50 | 14 | 11 | Pazeh | Taiwan | dbMHC | -0.12 | 0.50 | 1401 | 0.18 | -0.64 | [1, 2] |
| 62 | 46 | 12 | 12 | Atayal | Taiwan | dbMHC | -0.19 | 0.10 | 1401 | 0.09 | -0.64 | [1, 2] |
| 64 | 48 | 16 | 12 | Saisiat | Taiwan | dbMHC | 0.13 | 0.68 | 1401 | 0.10 | -0.64 | [1, 2] |
| 59 | 43 | 16 | 12 | Tsou | Taiwan | dbMHC | 0.04 | NA | 0803 | 0.12 | 0.06 | [1, 2] |
| 36 | 14 | 10 | 13 | China Yunnan Province Bai | China | AFND | -0.02 | 0.50 | 1202 | 0.37 | 1.58 | [31] |
| 63 | 47 | 14 | 13 | Siraya | Taiwan | dbMHC | -0.23 | 0.08 | 1401 | 0.09 | -0.64 | [1, 2] |
| 61 | 45 | 14 | 13 | Thao | Taiwan | dbMHC | 0.08 | 0.61 | 11011202 | 0.10 | -0.22 1.58 | [1, 2] |
| 20 | 21 | 17 | 14 | USA South Dakota Lakota Sioux | USA | AFND | -0.38 | NA | 0407 | 0.35 | -0.79 | [2] |
| 34 | 28 | 17 | 14 | USA South Dakota Sioux | USA | AFND | -0.42 | -0.17 | 0403 | 0.33 | -0.77 | [1, 2] |
| 38 | 30 | 28 | 15 | Bolivia Aymara | Bolivia | AFND | -0.47 | 0.18 | 0802 | 0.26 | -0.78 | [32] |
| 15 | 18 | 20 | 15 | Peru Titikaka Lake Uro | Peru | AFND | -0.54 | -0.54 | 0802 | 0.18 | -0.78 | [33] |
| 49 | 39 | 36 | 16 | Filipino | Philippines | dbMHC | -0.29 | 0.40 | 1502 | 0.21 | -0.77 | [1, 2] |
| 70 | 54 | 42 | 16 | Ivatan | Philippines | dbMHC | 0.04 | 0.76 | 1502 | 0.26 | -0.77 | [1, 2] |
| 31 | 26 | 15 | 17 | China Yunnan Province Jinuo | China | AFND | 0.23 | 0.83 | 1202 | 0.24 | 1.58 | [34] |
| 22 | 22 | 23 | 17 | Indonesia Sundanese and Javanese | Indonesia | AFND | 0.28 | 0.69 | 1202 | 0.56 | 1.58 | [2] |
| 13 | 16 | 18 | 18 | China Yunnan Province Han | China | AFND | 0.19 | 0.45 | 0901 | 0.16 | -0.51 | [35] |
| 14 | 17 | 19 | 19 | Mexico Oaxaca Mixtec | Mexico | AFND | -0.25 | NA | 0407 | 0.12 | -0.79 | [36] |
| 17 | 19 | 21 | 20 | Sweden Northern Sami | Sweden | AFND | -0.21 | 0.03 | 0801 | 0.24 | -0.21 | [15] |
| 19 | 20 | 22 | 21 | USA Arizona Gila River Amerindian | USA | AFND | 0.14 | NA | 1402 | 0.29 | 0.19 | [2] |
| 26 | 23 | 24 | 22 | China Yunnan Bulang | China | AFND | 0.71 | 1.06 | 1202 | 0.36 | 1.58 | [26] |
| 30 | 25 | 25 | 23 | Ecuador Cayapa | Ecuador | AFND | -0.46 | NA | 0407 | 0.17 | -0.79 | [37] |
| 32 | 27 | 26 | 24 | China Yunnan Province Wa | China | AFND | 0.20 | 0.69 | 1202 | 0.29 | 1.58 | [2] |
| 39 | 31 | 29 | 25 | Papua New Guinea South Gidra | Papua New Guinea | AFND | -0.28 | 0.67 | 1502 | 0.17 | -0.77 | [38] |
| 40 | 32 | 30 | 26 | Zulu | South Africa | dbMHC | -0.34 | -0.09 | 1101 | 0.16 | -0.22 | [1, 2] |
| 41 | 33 | 31 | 27 | Doggon | Mali | dbMHC | 0.10 | NA | 0804 | 0.32 | -0.17 | [1, 2] |
| 44 | 35 | 32 | 28 | Cape York | Australia | dbMHC | -0.23 | NA | 0803 | 0.15 | 0.06 | [1, 2] |
| 45 | 36 | 33 | 29 | Kimberley | Australia | dbMHC | -0.15 | NA | 0803 | 0.20 | 0.06 | [1, 2] |
| 46 | 37 | 34 | 30 | Seri | Mexico | dbMHC | -0.59 | NA | 0407 | 0.26 | -0.79 | [1, 2] |
| 47 | 38 | 35 | 31 | Lacandon | Mexico | dbMHC | -0.38 | NA | 0411 | 0.22 | -0.65 | [1, 2] |
| 53 | 40 | 37 | 32 | Yupik | Canada | dbMHC | -0.32 | 0.09 | 0401 | 0.28 | -0.54 | [1, 2] |
| 58 | 42 | 39 | 33 | Yami | Taiwan | dbMHC | -0.18 | 1.21 | 1401 | 0.18 | -0.64 | [1, 2] |
| 60 | 44 | 12 | 34 | Toroko | Taiwan | dbMHC | -0.30 | NA | 1401 | 0.12 | -0.64 | [1, 2] |
| 68 | 52 | 40 | 35 | Puyuma 49 | Taiwan | dbMHC | -0.31 | 0.39 | 1502 | 0.15 | -0.77 | [1, 2] |
| 69 | 53 | 41 | 36 | Bunun | Taiwan | dbMHC | 0.19 | 0.40 | 1202 | 0.20 | 1.58 | [1, 2] |
| 71 | 55 | 43 | 37 | Ami 97 | Taiwan | dbMHC | -0.52 | 0.32 | 0404 | 0.28 | -0.48 | [1, 2] |

**References**

1. J. A. Hansen H. Immunobiology of the human MHC - 13th IHWS Anthropology/Human Genetic Diversity Joint Report – Chapter 3: Population Reports.: IHWG Press; 2004.

2. Danver SL. Native peoples of the world: an Encylopedia of groups, cultures and contemporary issues: Routledge; 2015.

3. Saito S, Ota S, Yamada E, Inoko H, Ota M. Allele frequencies and haplotypic associations defined by allelic DNA typing at HLA class I and class II loci in the Japanese population. Tissue Antigens. 2000;56(6):522-9.

4. Kitawaki J, Obayashi H, Kado N, Ishihara H, Koshiba H, Maruya E, et al. Association of HLA class I and class II alleles with susceptibility to endometriosis. Hum Immunol. 2002;63(11):1033-8.

5. Yang JH, Sohn YH, Ko SY, Choi SE, Kim MH, Oh HB. Anthropological analysis of Koreans using HLA class II diversity among East Asians. Tissue Antigens. 2010;76(4):282-8.

6. Song EY, Park MH, Kang SJ, Park HJ, Kim BC, Tokunaga K, et al. HLA class II allele and haplotype frequencies in Koreans based on 107 families. Tissue Antigens. 2002;59(6):475-86.

7. Lee KW, Oh DH, Lee C, Yang SY. Allelic and haplotypic diversity of HLA-A, -B, -C, -DRB1, and -DQB1 genes in the Korean population. Tissue Antigens. 2005;65(5):437-47.

8. Hong SC, Lin L, Lo B, Jeong JH, Shin YK, Kim SY, et al. DQB1*0301 and DQB1*0601 modulate narcolepsy susceptibility in Koreans. Hum Immunol. 2007;68(1):59-68.

9. Song EY, Park H, Roh EY, Park MH. HLA-DRB1 and -DRB3 allele frequencies and haplotypic associations in Koreans. Hum Immunol. 2004;65(3):270-6.

10. Hsieh NK, Chu CC, Lee NS, Lee HL, Lin M. Association of HLA-DRB1*0405 with resistance to multibacillary leprosy in Taiwanese. Hum Immunol. 2010;71(7):712-6.

11. Riccio ME, Buhler S, Nunes JM, Vangenot C, Cuenod M, Currat M, et al. 16(th) IHIW: analysis of HLA population data, with updated results for 1996 to 2012 workshop data (AHPD project report). Int J Immunogenet. 2013;40(1):21-30.

12. Alfirevic A, Gonzalez-Galarza F, Bell C, Martinsson K, Platt V, Bretland G, et al. In silico analysis of HLA associations with drug-induced liver injury: use of a HLA-genotyped DNA archive from healthy volunteers. Genome Med. 2012;4(6):51.

13. Ronningen KS, Spurkland A, Markussen G, Iwe T, Vartdal F, Thorsby E. Distribution of HLA class II alleles among Norwegian Caucasians. Hum Immunol. 1990;29(4):275-81.

14. Kapustin S, Lyshchov A, Alexandrova J, Imyanitov E, Blinov M. HLA class II molecular polymorphisms in healthy Slavic individuals from North-Western Russia. Tissue Antigens. 1999;54(5):517-20.

15. Johansson A, Ingman M, Mack SJ, Erlich H, Gyllensten U. Genetic origin of the Swedish Sami inferred from HLA class I and class II allele frequencies. Eur J Hum Genet. 2008;16(11):1341-9.

16. Arnaiz-Villena A, Reguera R, Ferri A, Barbolla L, Abd-El-Fatah-Khalil S, Bakhtiyarova N, et al. The peopling of Madeira Archipelago (Portugal) according to HLA genes. Int J Immunogenet. 2009;36(1):9-14.

17. Spinola H, Bruges-Armas J, Mora MG, Middleton D, Brehm A. HLA genes in Madeira Island (Portugal) inferred from sequence-based typing: footprints from different origins. Mol Immunol. 2006;43(10):1726-8.

18. Begovich AB, Moonsamy PV, Mack SJ, Barcellos LF, Steiner LL, Grams S, et al. Genetic variability and linkage disequilibrium within the HLA-DP region: analysis of 15 different populations. Tissue Antigens. 2001;57(5):424-39.

19. Paximadis M, Mathebula TY, Gentle NL, Vardas E, Colvin M, Gray CM, et al. Human leukocyte antigen class I (A, B, C) and II (DRB1) diversity in the black and Caucasian South African population. Hum Immunol. 2012;73(1):80-92.

20. Muro M, Marin L, Torio A, Moya-Quiles MR, Minguela A, Rosique-Roman J, et al. HLA polymorphism in the Murcia population (Spain): in the cradle of the archaeologic Iberians. Hum Immunol. 2001;62(9):910-21.

21. Mahjoub S, Mehri S, Ghazouani E, Ouarda F, Boussada R, Zaroui A, et al. HLA class II polymorphisms in Tunisian patients with dilated cardiomyopathy. Tissue Antigens. 2010;75(6):679-83.

22. Petlichkovski A, Efinska-Mladenovska O, Trajkov D, Arsov T, Strezova A, Spiroski M. High-resolution typing of HLA-DRB1 locus in the Macedonian population. Tissue Antigens. 2004;64(4):486-91.

23. Arnaiz-Villena A, Dimitroski K, Pacho A, Moscoso J, Gomez-Casado E, Silvera-Redondo C, et al. HLA genes in Macedonians and the sub-Saharan origin of the Greeks. Tissue Antigens. 2001;57(2):118-27.

24. Hajeer AH, Al Balwi MA, Aytul Uyar F, Alhaidan Y, Alabdulrahman A, Al Abdulkareem I, et al. HLA-A, -B, -C, -DRB1 and -DQB1 allele and haplotype frequencies in Saudis using next generation sequencing technique. Tissue Antigens. 2013;82(4):252-8.

25. Pimtanothai N, Hurley CK, Leke R, Klitz W, Johnson AH. HLA-DR and -DQ polymorphism in Cameroon. Tissue Antigens. 2001;58(1):1-8.

26. Shi L, Shi L, Yao YF, Matsushita M, Yu L, Huang XQ, et al. Genetic link among Hani, Bulang and other Southeast Asian populations: evidence from HLA -A, -B, -C, -DRB1 genes and haplotypes distribution. Int J Immunogenet. 2010;37(6):467-75.

27. Chen S, Hu Q, Xie Y, Zhou L, Xiao C, Wu Y, et al. Origin of Tibeto-Burman speakers: evidence from HLA allele distribution in Lisu and Nu inhabiting Yunnan of China. Hum Immunol. 2007;68(6):550-9.

28. Lin JH, Liu ZH, Lv FJ, Fu YG, Fan XL, Li SY, et al. Molecular analyses of HLA-DRB1, -DPB1, and -DQB1 in Jing ethnic minority of Southwest China. Hum Immunol. 2003;64(8):830-4.

29. Ogata S, Shi L, Matsushita M, Yu L, Huang XQ, Shi L, et al. Polymorphisms of human leucocyte antigen genes in Maonan people in China. Tissue Antigens. 2007;69(2):154-60.

30. Shi L, Yao YF, Shi L, Matsushita M, Yu L, Lin QK, et al. HLA alleles and haplotypes distribution in Dai population in Yunnan province, Southwest China. Tissue Antigens. 2010;75(2):159-65.

31. Hu W, Tang L, Wang J, Wang B, Li S, Yu H, et al. Polymorphism of HLA-DRB1, -DQB1 and -DPB1 genes in Bai ethnic group in southwestern China. Tissue Antigens. 2008;72(5):474-7.

32. Arnaiz-Villena A, Siles N, Moscoso J, Zamora J, Serrano-Vela JI, Gomez-Casado E, et al. Origin of Aymaras from Bolivia and their relationship with other Amerindians according to HLA genes. Tissue Antigens. 2005;65(4):379-90.

33. Arnaiz-Villena A, Gonzalez-Alcos V, Serrano-Vela JI, Reguera R, Barbolla L, Parga-Lozano C, et al. HLA genes in Uros from Titikaka Lake, Peru: origin and relationship with other Amerindians and worldwide populations. Int J Immunogenet. 2009;36(3):159-67.

34. Shi L, Ogata S, Yu JK, Ohashi J, Yu L, Shi L, et al. Distribution of HLA alleles and haplotypes in Jinuo and Wa populations in Southwest China. Hum Immunol. 2008;69(1):58-65.

35. Yao Y, Shi L, Shi L, Matsushita M, Yu L, Lin K, et al. Distribution of HLA-A, -B, -Cw, and -DRB1 alleles and haplotypes in an isolated Han population in Southwest China. Tissue Antigens. 2009;73(6):561-8.

36. Hollenbach JA, Thomson G, Cao K, Fernandez-Vina M, Erlich HA, Bugawan TL, et al. HLA diversity, differentiation, and haplotype evolution in Mesoamerican Natives. Hum Immunol. 2001;62(4):378-90.

37. Trachtenberg EA, Erlich HA, Rickards O, DeStefano GF, Klitz W. HLA class II linkage disequilibrium and haplotype evolution in the Cayapa Indians of Ecuador. Am J Hum Genet. 1995;57(2):415-24.

38. Yoshida M, Ohtsuka R, Nakazawa M, Juji T, Tokunaga K. HLA-DRB1 frequencies of non-Austronesian-speaking Gidra in south New Guinea and their genetic affinities with Oceanian populations. Am J Phys Anthropol. 1995;96(2):177-81.
