# Supplementary material for: Pathogen diversity drives the evolution of generalist MHC-II alleles in human populations
Source: PLoS Biol. 2019 Jan 31;17(1):e3000131. doi: 10.1371/journal.pbio.3000131 (PMC6372212; doi:10.1371/journal.pbio.3000131)
Supplement: S2 Table — (DOCX) [file pbio.3000131.s011.docx]

**S2 Table. The relationship between promiscuity and pathogen diversity is independent of HLA diversity and country size.** We tested the effects of potential confounding factors, such as HLA genetic diversity and country size, on the association between promiscuity and extracellular pathogen diversity in a multivariate linear regression model. We also explicitly tested whether the effect of extracellular pathogen diversity remains when controlling for intracellular pathogen diversity. To this end, we added several predictor variables to our model as follows:

$promiscuity \sim ic div+ec div+HLA div+country size$**,**

where *ic div* is intracellular pathogen diversity, *ec div* is extracellular pathogen diversity, *HLA* *div* is average HLA diversity in the given population group, and *country size* is the average of country size values where populations are localized in km^2^. The relationship between extracellular pathogen diversity and promiscuity remained significant after controlling for these potential confounding factors and intracellular pathogen diversity. There was non- significant or very weak (explained variance is 0.007) negative relationship between intracellular pathogen diversity and promiscuity when extracellular pathogen diversity was accounted for. HLA diversity of countries were compiled from a recent paper [1]. Country size data were downloaded from World Bank DataBank (<http://databank.worldbank.org>). Population groups were created using 15^th^ percentile genetic distance cutoff (see Methods). For results of HLA class I loci and results when using different distance cutoff values, see S3 Data.

|  | **promiscuity ~ ic div + ec div + HLA div + country size** | | | | | | | |
| --- | --- | --- | --- | --- | --- | --- | --- | --- |
|  | **Predicted promiscuity** | | | | **In vitro promiscuity** | | | |
|  | **Variable** | **Slope** | **Variance explained** | **P-value** | **Variable** | **Slope** | **Variance explained** | **P-value** |
|  | **ic div** | - | 0.007 | 0.04 | **ic div** | - | 0.04 | 0.22 |
|  | **ec div** | + | 0.29 | 1*10^-4^ | **ec div** | + | 0.47 | 1*10^-4^ |
|  | **HLA div** | - | 0.02 | 0.65 | **HLA div** | - | 0.07 | 0.09 |
|  | **country size** | + | 0.12 | 0.02 | **country size** | - | 0.001 | 0.81 |
| **R^2^** | **0.36** | | | | **0.51** | | | |
| **N** | **37** | | | | **28** | | | |
| **P-value of BP test for heterosc.** | **0.39** | | | | **0.47** | | | |

Significant relationships between promiscuity and the indicated variables are highlighted with red color. ic div: intracellular pathogen count; ec div: extracellular pathogen count; HLA div: expected heterozygosity of HLA from [2]; P-value indicates the probability of observing a relationship between the predictor and response variables due to chance R^2^: Total variance in promiscuity explained by the model (adjusted for the number of variables). N: the number of population groups. Non-significant P-value of Breusch-Pagan (BP) test indicates lack of heteroscedasticity.

**References**

1. Cook CJ. The natural selection of infectious disease resistance and its effect on contemporary health. Review of Economics and Statistics. 2015;97(4):742-57.

2. Fedderke JW, Klitgaard RE, Napolioni V. Genetic adaptation to historical pathogen burdens. Infect Genet Evol. 2017;54:299-307.
