# Supplementary material for: Pathogen diversity drives the evolution of generalist MHC-II alleles in human populations
Source: PLoS Biol. 2019 Jan 31;17(1):e3000131. doi: 10.1371/journal.pbio.3000131 (PMC6372212; doi:10.1371/journal.pbio.3000131)
Supplement: S3 Table — (DOCX) [file pbio.3000131.s012.docx]

**S3 Table. Results of HLA association studies suggest a protective role of high allele promiscuity in infectious diseases.** Diseases caused by intracellular pathogens only were excluded from the analysis. Each row refers to one disease or group of several diseases, like opportunistic infections in AIDS. HLA-disease associations were classified into detrimental (Detr.), controversial (Con.) or beneficial (Ben.) groups based on the results of systematic literature search. In each allele group, the number of detrimental and beneficial associations were determined. Controversial associations were excluded from the analysis. Detrimental associations were significantly underrepresented, while beneficial associations were significantly overrepresented in the promiscuous allele group (DRB1*12) compared to the non-promiscuous one (DRB1*03) (Fisher exact test P: 0.003). For detailed results of the systematic literature search, see S5 Data.

| **Disease** | **DRB1*12** | | | **DRB1*03** | | |
| --- | --- | --- | --- | --- | --- | --- |
|  | **Detr.** | **Con.** | **Ben.** | **Detr.** | **Con.** | **Ben.** |
| Typhoid fever |  |  | x |  |  |  |
| Lyme's disease |  |  |  | x |  |  |
| Tuberculosis |  |  | x |  |  | x |
| H. pylori infection |  | x |  |  | x |  |
| Leprosy |  |  | x |  | x |  |
| Periodontal infections |  |  | x |  |  |  |
| Schistosomiasis |  |  | x | x |  |  |
| S. aureus (carriage) |  |  |  | x |  |  |
| H. influenzae |  |  |  | x |  |  |
| E. granulosus |  |  |  | x |  |  |
| Amebic liver abscess |  |  |  | x |  |  |
| Onchocerciasis |  |  |  | x |  |  |
| Opportunistic infections in AIDS |  |  |  | x |  |  |
| **Total number** | 0 | 1 | 5 | 8 | 2 | 1 |
