# Supplementary material for: Pathogen diversity drives the evolution of generalist MHC-II alleles in human populations
Source: PLoS Biol. 2019 Jan 31;17(1):e3000131. doi: 10.1371/journal.pbio.3000131 (PMC6372212; doi:10.1371/journal.pbio.3000131)
Supplement: S1 Text — (DOCX) [file pbio.3000131.s013.docx]

**S1 Text. Pathogen diversity and promiscuity of HLA class I alleles.**

Our hypothesis predicts that promiscuous HLA alleles should reside in geographical regions of high pathogen diversity, as they confer a broader recognition of epitopes at the individual level. However, other unrelated evolutionary forces may shape the geographical distribution of promiscuous alleles. For example, the HLA-C locus has exceptionally important functions unrelated to pathogen epitope recognition and presentation [1].

We found positive correlation between local intracellular pathogen diversity and the HLA-A promiscuity level of the corresponding human populations (S9 Fig, A, S3 Data). At best, a marginally significant positive correlation was found for HLA-B (S9 Fig, C, S3 Data), and no correlation at all for HLA-C (S9 Fig, E, S3 Data). HLA-C has a relatively low abundance on the surface of infected cells compared to HLA-A and HLA-B [2]. This could be a prerequisite of NK cell mediated recognition of infected cells [1]. Accordingly, HLA-C has a relatively minor role in presenting intracellular pathogen-derived epitopes. Indeed, existing data indicate that only a relatively small number of peptide epitopes can bind HLA-C, compared to HLA-A and HLA-B [3].

The reason for the weak association between HLA-B promiscuity level and pathogen richness is much less clear. Two complementary population genetic mechanisms - pathogen-driven balancing selection (PDBS) and selection for elevated promiscuity – are expected to act in regions of high pathogen load. We speculate that under strong balancing selection of HLA alleles, selection on elevated population promiscuity level may be dampened, as highly divergent allele pairs in heterozygous individuals can bind epitopes of more pathogen species [4]. The population genetic analysis of multiple HLA loci supports this argument: especially strong balancing selection operates at the HLA B locus [5]. This hypothesis will be explored in detail in a future work.

**References**

1. Parham P, Norman PJ, Abi-Rached L, Guethlein LA. Human-specific evolution of killer cell immunoglobulin-like receptor recognition of major histocompatibility complex class I molecules. Philos Trans R Soc Lond B Biol Sci. 2012;367(1590):800-11.

2. Kulkarni S, Savan R, Qi Y, Gao X, Yuki Y, Bass SE, et al. Differential microRNA regulation of HLA-C expression and its association with HIV control. Nature. 2011;472(7344):495-8.

3. Kaufman J. Generalists and Specialists: A New View of How MHC Class I Molecules Fight Infectious Pathogens. Trends Immunol. 2018;39(5):367-79.

4. Pierini F, Lenz TL. Divergent allele advantage at human MHC genes: signatures of past and ongoing selection. Mol Biol Evol. 2018.

5. Satta Y, O'HUigin C, Takahata N, Klein J. Intensity of natural selection at the major histocompatibility complex loci. Proc Natl Acad Sci U S A. 1994;91(15):7184-8.
